# Improving 3D photogrammetry models through spectral imaging: Tooth enamel as a case study

**DOI:** 10.1371/journal.pone.0220949

**Published:** 2019-08-13

**Authors:** Aurore Mathys, Patrick Semal, Jonathan Brecko, Didier Van den Spiegel

**Affiliations:** 1 Biological Collection and Data Management, Royal Museum for Central Africa, Tervuren, Belgium; 2 Scientific Heritage Service, Royal Belgian Institute of Natural Sciences, Brussels, Belgium; Universidade Federal de Uberlandia, BRAZIL

## Abstract

Reflective or translucent materials are a challenge to digitize in 3D. Results are better with a matt coating although objects from museum collections are often too fragile or too valuable to be treated in this way. It is therefore essential that alternative solutions are found. This study analyzed spectral photogrammetry as a possible solution. Spectral photogrammetry is an emerging technique which uses images at different wavelengths to create 3D models. Tooth enamel is a challenging material to digitize. Six sets of teeth were photographed at different wavelengths. The results showed that the quality of the models enamels parts improved when taken with ultraviolet wavelengths whilst models were less accurate when photogrammetry was performed with the red and infrared spectrum. This can be explained by the optical properties of enamel. This study demonstrates that knowing the optical properties of a material beforehand could help future photogrammetric digitization of challenging materials.

## Introduction

3D digitization is nowadays frequently used in a museum environment for research [1–4], conservation [5–8] or dissemination [9–12]. In recent years more and more initiatives have been started in order to investigate the possibility of using the combination of spectral and 3D imaging for cultural heritage. 3D spectral imaging has previously focused on things such as how 3D spectral imaging can enhance features [13–14], identify materials [13–15], record the spectral reflectance [15–17], produce more accurate colors of the object [18], estimate condition/deterioration [14, 19–22], but not on improving the quality of the 3D model.

There are different methods to attain the 3D digitization of objects in museums and these include CT imaging, structured light scanner and photogrammetry. Photogrammetry is commonly used as it is a low cost and versatile technique to obtain 3D models [23]. However, some materials are not well rendered with white light photogrammetry. Homogeneous (featureless) surfaces, such as plaster casts or objects in ivory, are not recorded with a high level of detail [24]. Highly reflective surfaces, such as polished metals or varnished ceramics, tend to generate a lot of “noise” (see definition in Method section) on the model [25]. Previous studies on digitization suggest that to accurately scan transparent, shiny or reflective objects, they should be coated with something (developer spray, anti-glare spray, dulling spray, chalk spray, cyclododecane spray, talc dust, paint, etc.) to reduce the reflections [26–28]. However, this isn’t possible for most museum collection items due to the removal process of the coating or the presence of chemical compounds in the coating spray that can interact with the object (like acetone) [29], therefore an alternative is necessary. The hypothesis that the integration of photogrammetry and multispectral imaging could help to improve recording the surface of these challenging objects is the main focus of this research.

A material that is widely common in both archaeology (anthropological remains) and natural sciences (vertebrates) collections is enamel (teeth). Like plaster, ivory etc, enamel is one such difficult material to capture with traditional 3D digitization techniques such as classic photogrammetry or laser scans [30]. This is largely due to the fact it has a reflective white featureless texture [31] and is translucent [32]. Previous work on photogrammetry, applied on modern samples, proposes that the best method to accurately capture enamel is to paint the surface with an opaque texture paint [31]. However, this cannot be applied to the fragile remains part of the museum collections. Therefore this material is an excellent model to test our hypothesis.

## Material

Teeth are present everywhere in our collections: They are abundant in both anthropological collections as they are in vertebrates collections. In the case of anthropological collections, teeth are an excellent object from which to extract DNA. As DNA extraction is a partially destructive analysis, it is essential to digitize them in 3D beforehand to document the complete specimen surface geometry.

**Fig 1:**
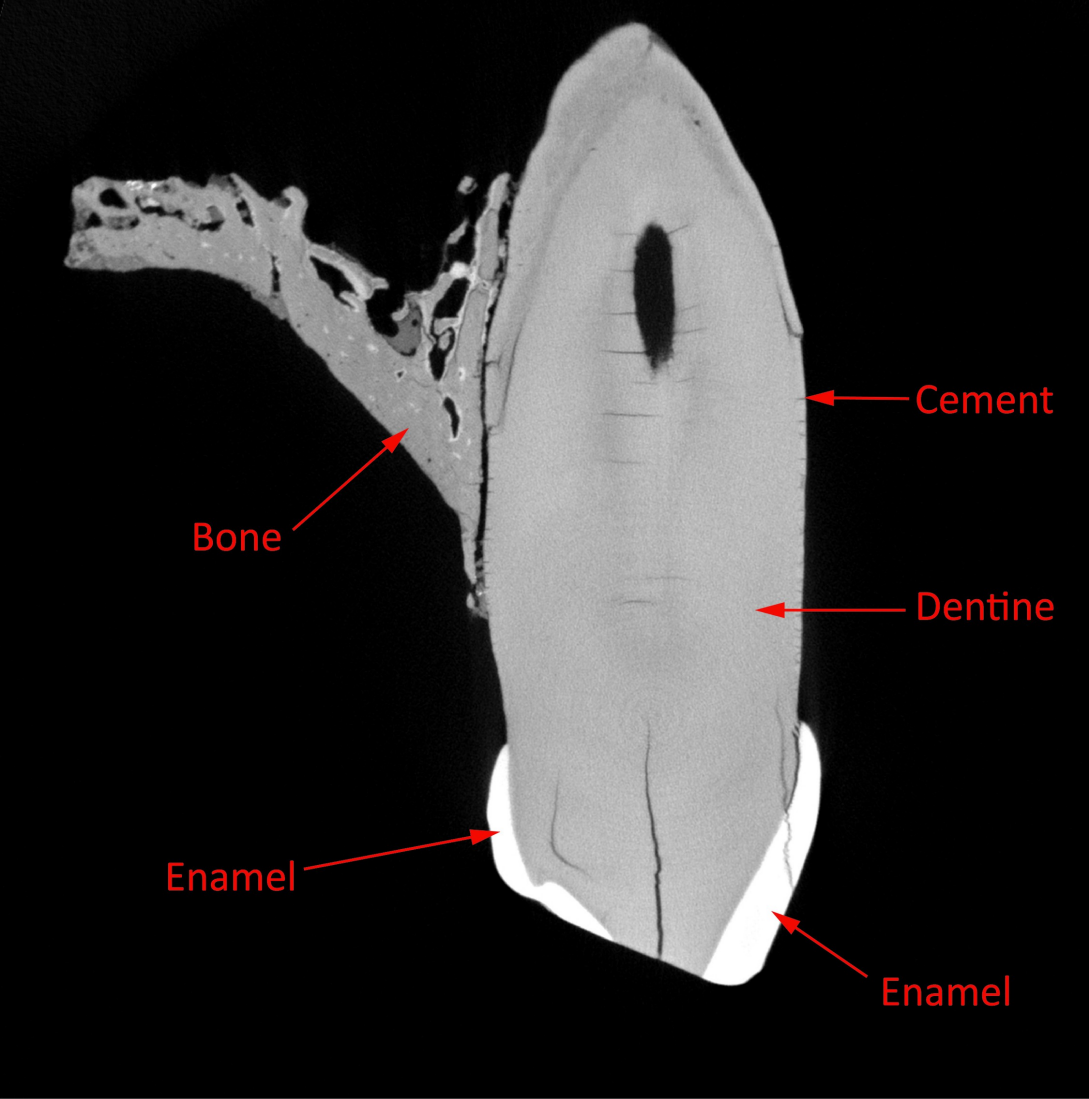
Structure of a tooth. Structure of a tooth of Spy 2B (RBINS) in X-Ray digitized with the RX Solution EasyTom 150 (22μm voxel size).

The external surface of a tooth is composed of two main parts: enamel and cementum (Fig 1). It is the enamel which is challenging to digitize as it is a crystalline reflective material composed mainly of hydroxyapatite (Ca10 (PO4)6. 2OH) [33].

This study used two Neanderthal maxillary fragments with teeth which were found in the Spy cave in Belgium (Spy 2A and Spy 2B) [34] and a partial mandible of a modern human from the necropolis of the abbey of Koksijde (BE) [35]. All the human remains are part of archaeological excavations of Belgian sites and belong to the collections of the Royal Belgian Institute of Natural Sciences (RBINS).

To show that the method is not limited to human enamel, the same technique was applied to different vertebrates: a hyena *(Hyaena* sp.*)*, a lion (*Panthera leo)* and a leopard (*Panthera pardus)* from the Royal Museum for Central Africa (RMCA) collections.

## Method

### Spectral photogrammetry

**Fig 2:**
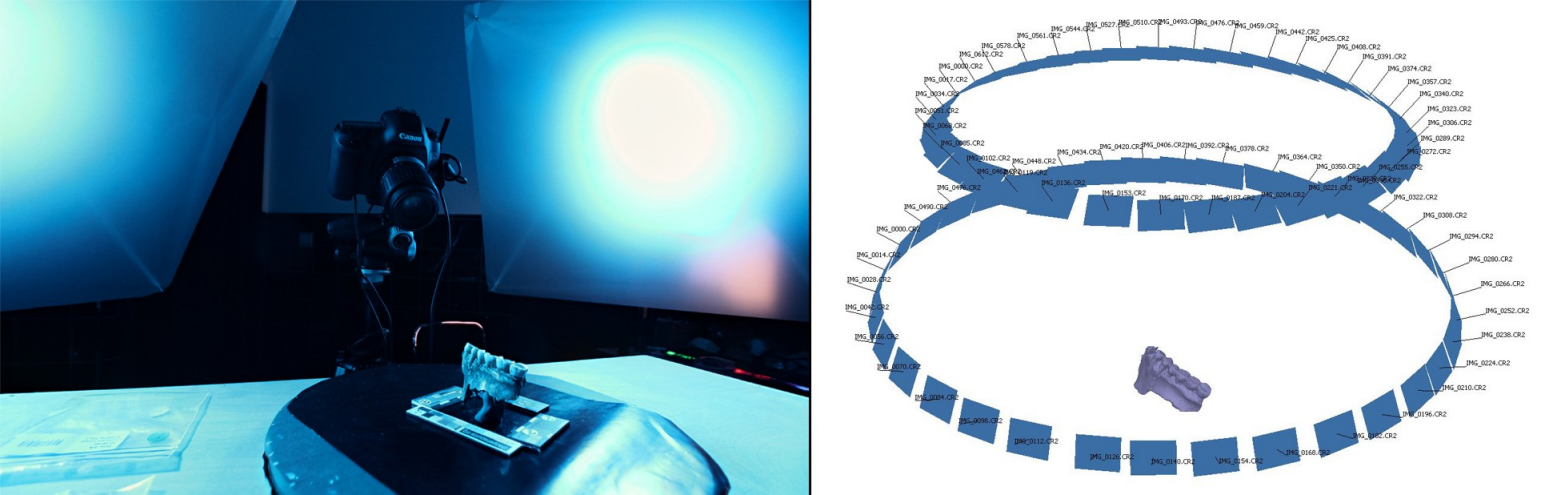
Photogrammetry setup (Specimen Spy 2A on a rotating table with scales and photogrammetry coded targets, and light panels) on the left; result of a typical camera network in a photogrammetry reconstruction software on the right.

The different specimens were digitized by multispectral photogrammetry. Photogrammetry is a technique that allows 3D reconstruction of surface geometry from photographs of the same object taken from multiple views (Fig 2). Multispectral imaging consists of taking images at different wavelengths. In order to perform multispectral photogrammetry, two modified DSLRs were used: a Canon 600D and a Canon 5Ds. A modified DSLR is a camera where the infrared (IR) cut-off filter in front of the sensor has been removed in order to allow visible spectrum and IR radiations to pass. This is called a full-spectrum conversion [36]. Modified cameras are also more sensitive to ultraviolet light (UV). A modified DSLR was chosen instead of a dedicated multispectral camera because these have a lower resolution compared to modern DSLRs and are also usually more expensive. The resolution of the digital image is very important to obtain good photogrammetry models. In addition to this, the modified DSLR has a sensitivity in both near UV and a portion of near infrared, while many multispectral dedicated cameras have sensitivity limited to either visible spectrum and IR or visible spectrum and UV.

The modified DSLR was combined with a Coastal Optic 60 mm macro lens (https://www.jenoptik-inc.com/) because it is an apochromatic macro lens between 310 and 1100 nm. This mean it is not necessary to refocus the lens when using different wavelengths. It is a lens made of quartz and fluorite instead of a glass lens, enabling more UV light to pass through.

**Fig 3:**
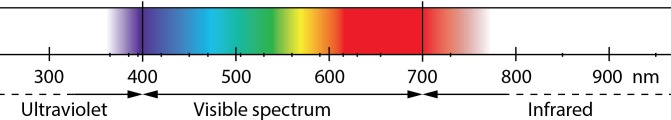
Spectrum of interest (nm).

To illuminate the object, LED lights with 15 different wavelengths were used (365nm, 385nm, 395nm, 420nm, 450nm, 470nm, 505nm, 530nm, 560nm, 590nm, 615nm, 630nm, 655nm, 735nm, 850nm, 950nm; Mega-vision system) and white light (Fig 3). The advantages of LED light is they are generating less heat than halogen lights, this is valuable for fragile material that could be damaged by changes in temperature as spectral photogrammetry acquisition can be time-consuming.

**Fig 4:**
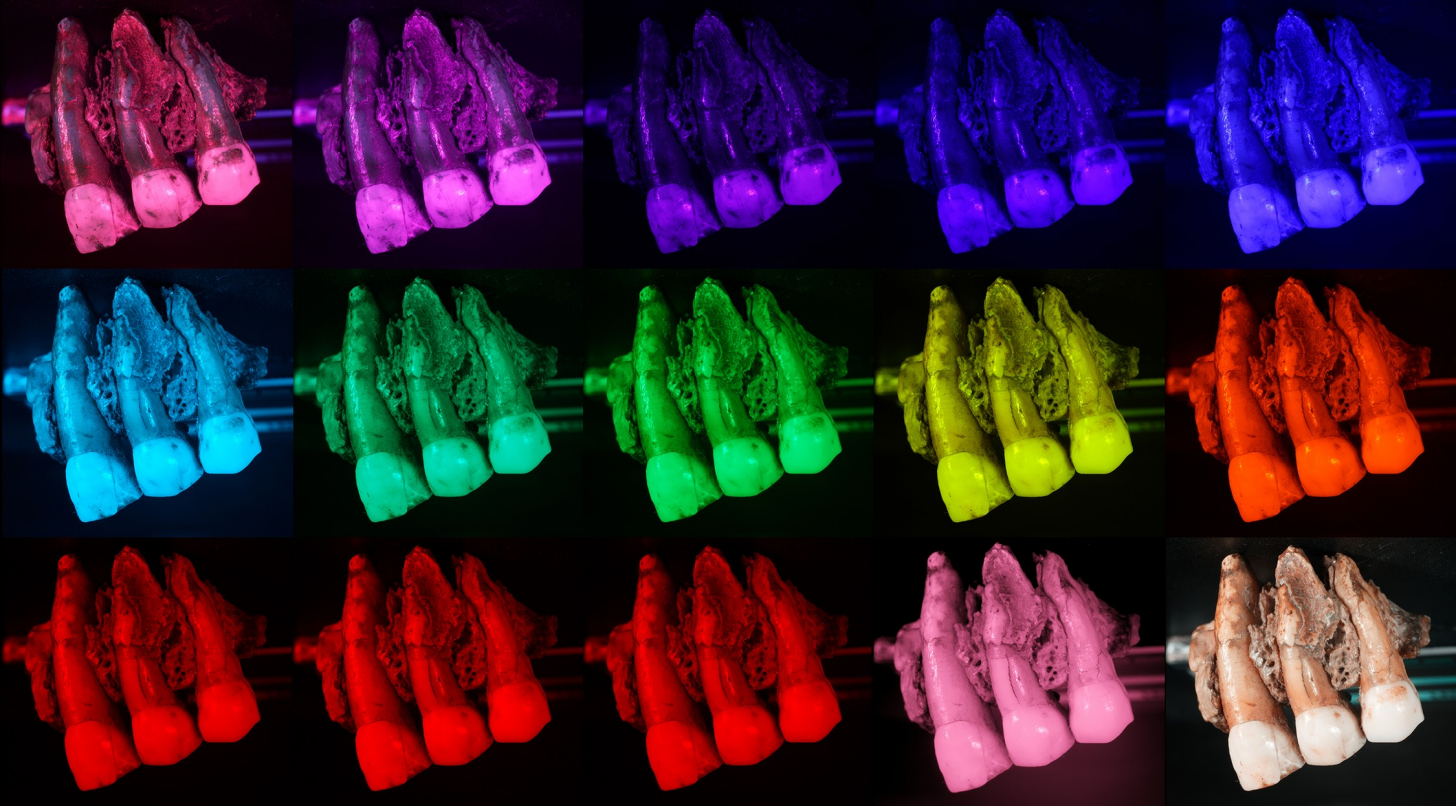
Pictures of Spy 2B at the different wavelengths. No filter. First row (left to right): 365 nm, 385 nm 395 nm, 420 nm, 450 nm. Second row (left to right): 470 nm, 505 nm, 530 nm, 560 nm, 590 nm. Third row (left to right): 615 nm, 630 nm, 655 nm, 850 nm, white light.

**Fig 5:**
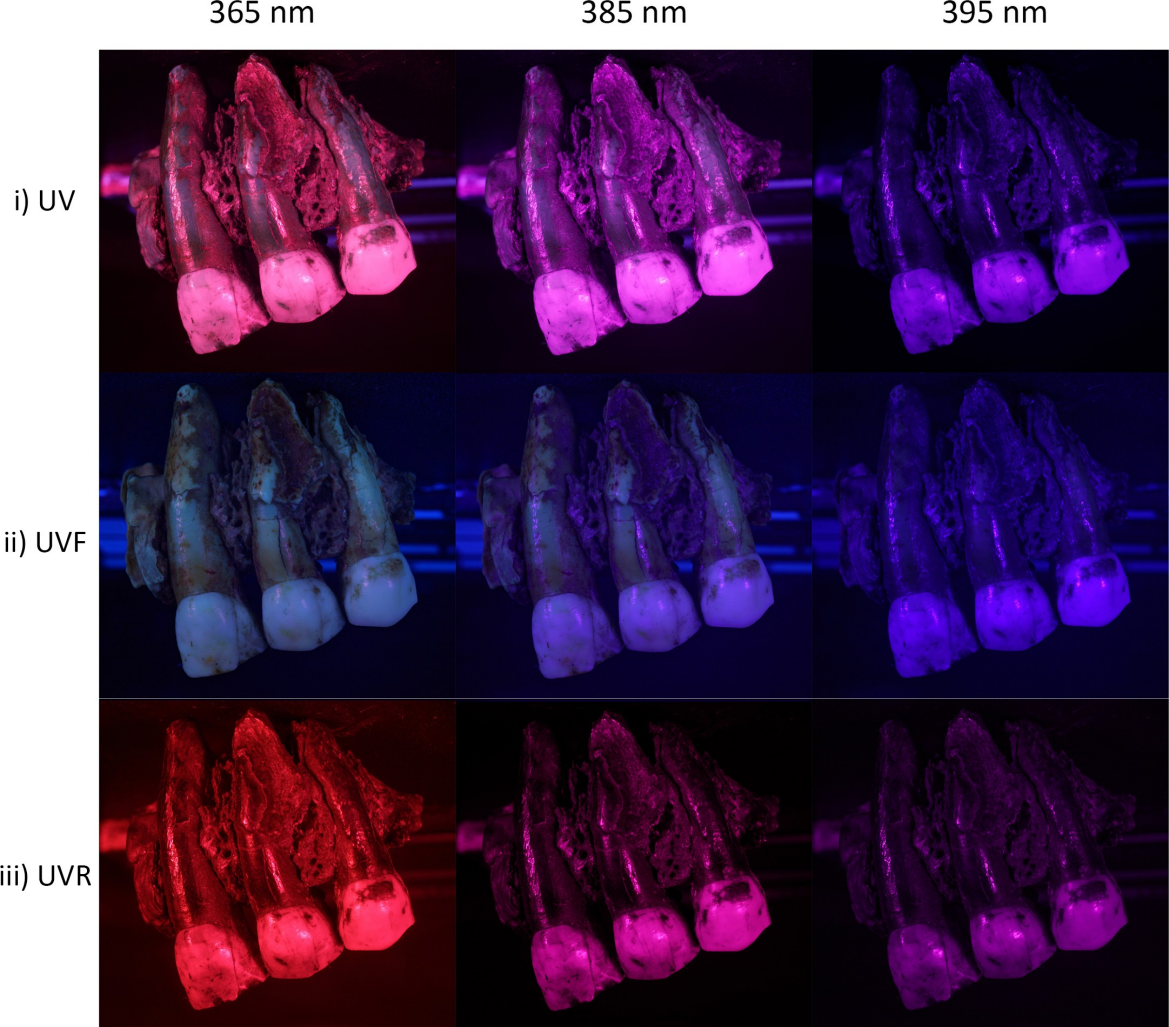
Differences between images of Spy 2B captured in UV, UVF and UVR. i) (left to right): 365 nm, 385 nm, 395 nm without filter (therefore both reflectance and fluorescence are captured). ii) UV fluorescence captured at 365 nm, 385 nm, 395 nm using a UV cut-off filter. iii) UV reflected at 365 nm, 385 nm, 395 nm with a UV-pass filter (395 nm is under the half bandwidth of the UV pass filter transmit, therefore not much UV reflectance is captured).

Photographs taken with the different wavelengths and white light (covering the full reflected spectrum) were made without a filter (Fig 4). Photographs in UV were made using: (i) no filter, capturing both fluorescence and reflected UV; (ii) a UV-cut filter (Baader UV/IR Cut / L-Filter, HBW 420–680 nm) in order to capture just fluorescence (UVF); (iii) a UV-pass filter (Baader U-Filter, HBW 320–380 nm) that lets only UV wavelengths pass through the lens in order to capture UV reflectance (UVR) exclusively (Fig 5). Finally, photographs were made with a polarizing filter (Marumi EXUS circular PL) for a few selected wavelengths. Details of the wavelengths and filters used for each photograph are available in Table 1. The camera position remains the same for capturing in each wavelength. Then the rotating table is rotated 10° to capture the next images for all the wavelengths. The exposure time for each wavelength is adjusted in order to have a correctly lit picture. Photogrammetry coded targets and scales are present on the pictures in order to align and scale the photogrammetry models (Fig 2).

**Table 1:** Photogrammetry capture parameters.

Specimens	N° of pictures	Light/Wavelengths	Filters
**Spy 2A** Canon 600D (18 MP APS-C *sensor)*	144	White	
**Spy 2A** Modified Canon 600D (18 MP APS-C *sensor)*	72 & 144	365, 385, 395, 420, 450, 470, 505, 530, 560, 590, 615, 630, 655, 735, 850, 950, white	
	72	365	UV-pass filter (UVR)
	72	385, white	Polarizing filter
**Spy 2A** Modified Canon 5Ds (50 MP full frame *sensor)*	72	365, 385, 395, 420, 450, 470, 505, 530, 560, 590, 615, 630, 655, 735, 850, 950, white	
	72	365, 385, 395	UV-pass filter (UVR)
	72	365, 385, 395	UV-IR cut filter (UVF)
**Spy 2B** Modified Canon 5Ds	72	365, 385, 395, 420, 450, 470, 505, 530, 560, 590, 615, 630, 655, 735, 850, 950, white	
	72	365, 385, 395	UV-pass filter (UVR)
	72	365, 385, 395	UV-IR cut filter (UVF)
**Koksijde** Modified Canon 5Ds	71	365, 385, 395, 420, 450, 470, 505, 530, 560, 590, 615, 630, 655, 735, 850, 950, white	
**Hyena *(Hyaena* sp*)*** Modified Canon 5Ds	72	365, 385, 395, 420, 450, 470, 505, 530, 560, 590, 615, 630, 655, 735, 850, 950, white	
	72	365, 385, 395	UV-pass filter (UVR)
	72	365, 385, 395	UV-IR cut filter (UVF)
**Lion *(Panthera leo)*** Modified Canon 5Ds	108	365, 385, 395, 420, 450, 470, 505, 530, 560, 590, 615, 630, 655, 850, 950, white	
	108	365, 385, 395	UV-pass filter (UVR)
	108	365, 385, 395	UV-IR cut filter (UVF)
**Leopard *(Panthera pardus)*** Modified Canon 5Ds	108	365, 385, 395, 420, 450, 470, 505, 530, 560, 590, 615, 630, 655, 850, 950, white	
	108	365, 385, 395	UV-pass filter (UVR)
	108	365, 385, 395	UV-IR cut filter (UVF)

List of all the specimens capture with the number of pictures, the wavelengths and the filters used. The detail protocol is available at dx.doi.org/10.17504/protocols.io.zzgf73w.

Spy 2A was digitized with both modified cameras. The other specimens were digitized only with the modified Canon 5Ds.

Models were created in a photogrammetry software used for image reconstruction (Agisoft PhotoScan Pro 1.4.2, http://www.agisoft.com/) from the untreated Canon raw (.cr2) pictures for each wavelength.

### Other 3D digitization techniques used

In addition to that, in order to compare the spectral photogrammetry with classic 3D techniques, Spy 2A was digitized with a medical Computed Tomography (CT), a micro-Computed Tomography (μCT), a structure light (SL) scanner, a triangulation laser scanner (TL) and classic photogrammetry (Ptg 600D - 1). Spy 2B was also digitized with a μCT scanner.

Spy 2A and Spy 2B were digitized with μCT using an RX Solutions EasyTom 150, at respectively 50 μm and 22 μm voxelsize. The CT was acquired with a Siemens Sentation 64 at 222 μm voxelsize. For both μCT and CT, the segmentation and surface reconstruction were performed using Dragonfly software Version 3.5 for Windows (Object Research Systems (ORS) Inc, Montreal, Canada, 2018; software available at http://www.theobjects.com/dragonfly). The 3D models obtained present staircasing errors (scanning artefact) due to the inter slice distance of the data, therefore the extracted mesh must be smoothed to a certain degree for an accurate surface representation [37–38]. The models were smoothed with an amount of five iterations with the default smoothing algorithm of Dragonfly.

The structured light model was obtained from and HDI Advance R3X at a theoretical resolution of 124/248μm. The laser scanner model was obtained from a NextEngine. NextEngine can achieve a theoretical resolution of 127 μm. The classic photogrammetry model was captured with an unmodified Canon 600D with white light.

### Evaluation methodology

3D models of different techniques and of different photogrammetry wavelengths are aligned using a certified inspection software (GOM Inspect, https://www.gom.com/3d-software/gom-inspect.html, freeware) best-fit alignment algorithms. Then, they are compared qualitatively and with surface comparison using GOM Inspect and CloudCompare (https://www.danielgm.net/cc/).

The qualitative evaluation is performed through the observation of the amount of noise and outliers present on the surfaces. Artefacts or noise are errors or aberrations in the data [39–40]. Outliers are discordant data or anomalies, it is data situated at an abnormal distance from the rest. An outlier is a form of heavy noise [41–43].

The surface deviation is measured as the distance between two models.

Specimen Spy 2A was compared to other digitization techniques while the rest of the models are only compared to each other.

## Results

All the photogrammetry data sets are processed with the same version of Agisoft Photoscan (1.4.2). Different amounts of photographs and rotation combinations were used for each datasets (Table 1).

The first part of the analysis focuses on Spy 2A in order to compare spectral photogrammetry results between them and to other digitization technique. The second part of the analysis aim to confirm or refute the spectral photogrammetric results. The quality of the models is evaluated using surface deviation and qualitative assessment.

### Spy 2A

Spy 2A was digitized with one medical CT, one μCT, a structured light scanner, a triangulation laser scanner and several photogrammetry using a Canon 600D (Ptg 600D - 1), a modified Canon 600D (Ptg 600D - 2) and a modified Canon 5Ds (Ptg 5Ds).

The differences between the models were evaluated using geometric deviation values, standard deviation and qualitative assessment. In order to proceed to this evaluation, one of the model has to be set as a reference (nominal geometry). As there is no ground truth for organic objects, the μCT model was set as nominal geometry as it is considered the more accurate as optical properties of the material do not interfere and the machine is certified to industry standard. The model obtained with μCT has a voxel size of 50 μm and has been segmented semi-automatically in Dragonfly.

The models were all aligned to the μCT model in GOM Inspect with a “best-fit” algorithm (a variant of ICP). The geometric deviation values and standard deviation were obtained in CloudCompare with an algorithm calculating the point distance to the reference model. The distribution of those values was displayed as a scalar field (red and blue represents the maximum deviation while green represent the parts where there is minimal deviation). The geometric deviation visualization enables us to see quickly where the larger deviations are located.

#### General comparison of surface deviation of the different models

**Fig 6:**
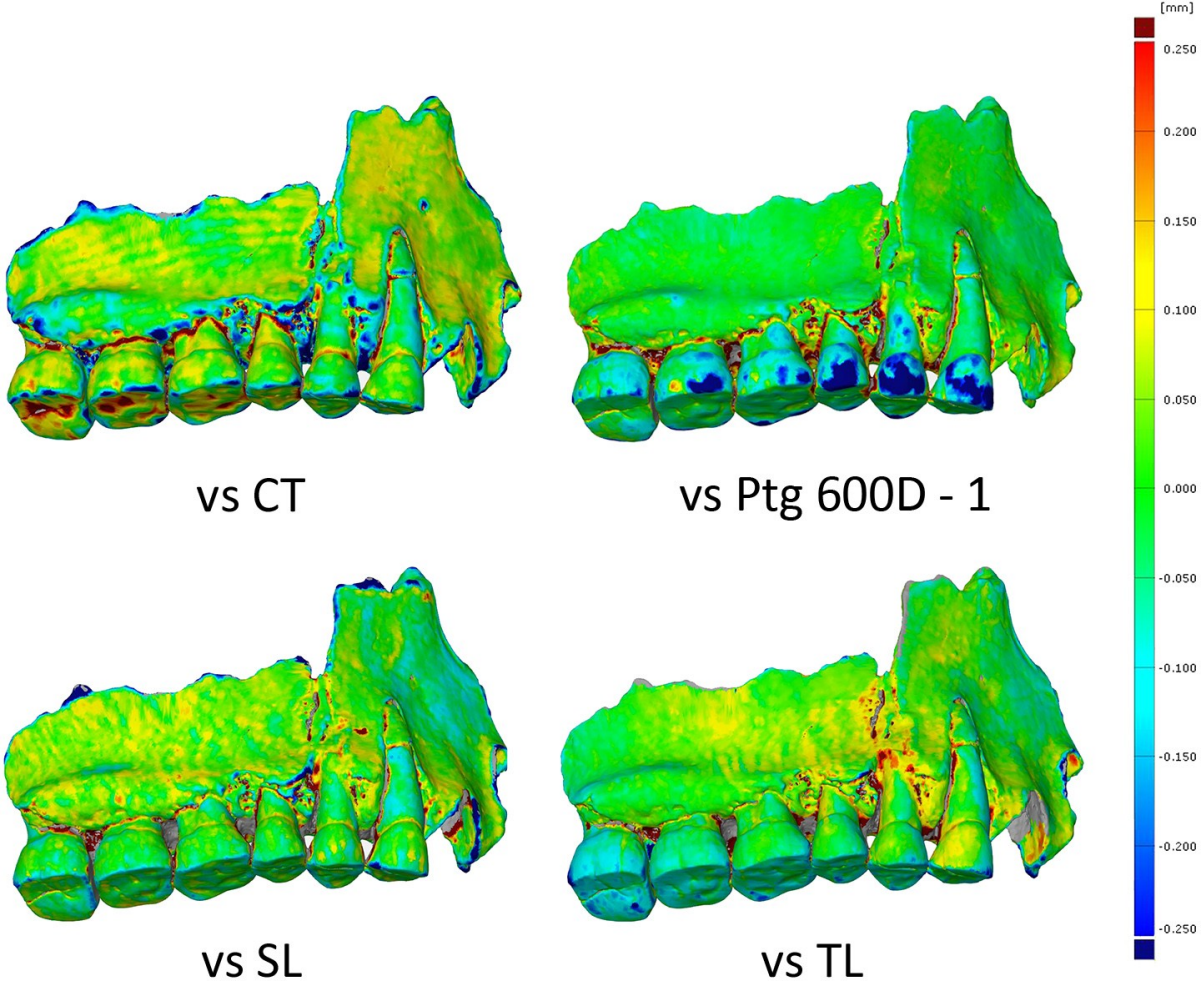
Scalar field representing the deviation between 3D models obtained with different classic 3D digitization techniques.

**Fig 7:**
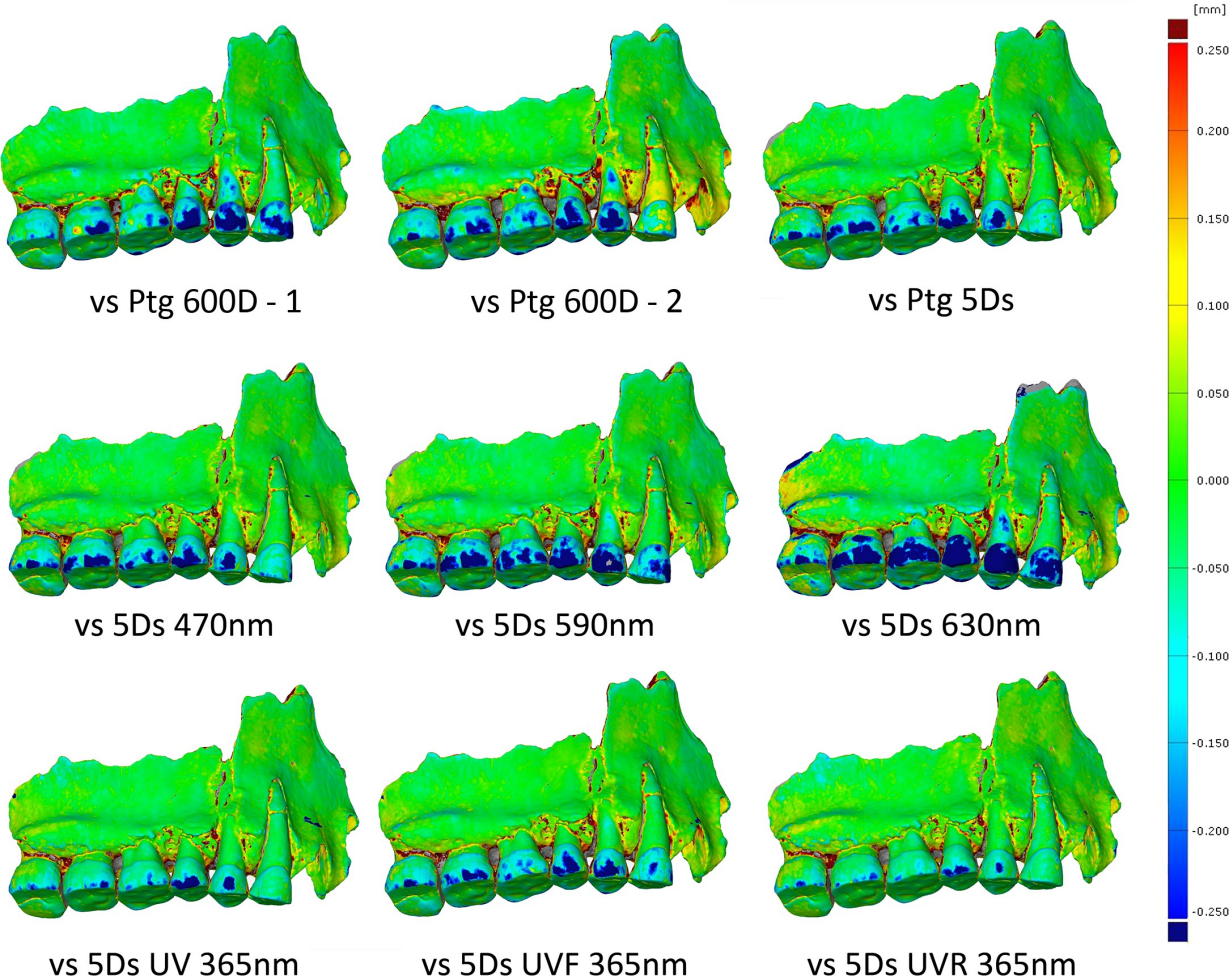
Scalar field representing the deviation between 3D models obtained with photogrammetry at different wavelengths. First row: 3 different photogrammetry models in white light with 3 different DSLR’s and made by 2 persons. Second row: Models at 3 different wavelengths in the visible spectrum. Third row: Models made under UV exposure combine to different filters to separate fluorescence and reflectance.

First, we looked at the differences between the models obtained with different techniques. The comparison with medical CT model shows scanning artefacts from the slice distance even though the model has been smoothed. The structured light model (SL) shows good quality enamel but differences on the bone surface. The laser model (TL) shows discrepancies on both enamel and bone. The white light photogrammetry model shows differences of more than 250 μm on parts of the enamel (Fig 6). Second, we compared the visualization of the geometric deviation between the different white light photogrammetry models and we were able to observe that the three models showed similar deviation on enamel on all models (Fig 7). Finally, we compared the deviation between the different wavelengths: from the deviation scalar fields we can see that the best results were obtained in UV and the models were getting less accurate as the wavelengths were longer. Models obtained in white light presented more deviation than the models in UV, but are more accurate than the models obtained in the yellow and red wavelengths. The models between UV, UVF and UVR are also compared and results showed that models obtained in UVF showed larger deviation. The model captured in UVR is the best, but the difference between UVR and UV seemed very small (Fig 7).

**Fig 8:**
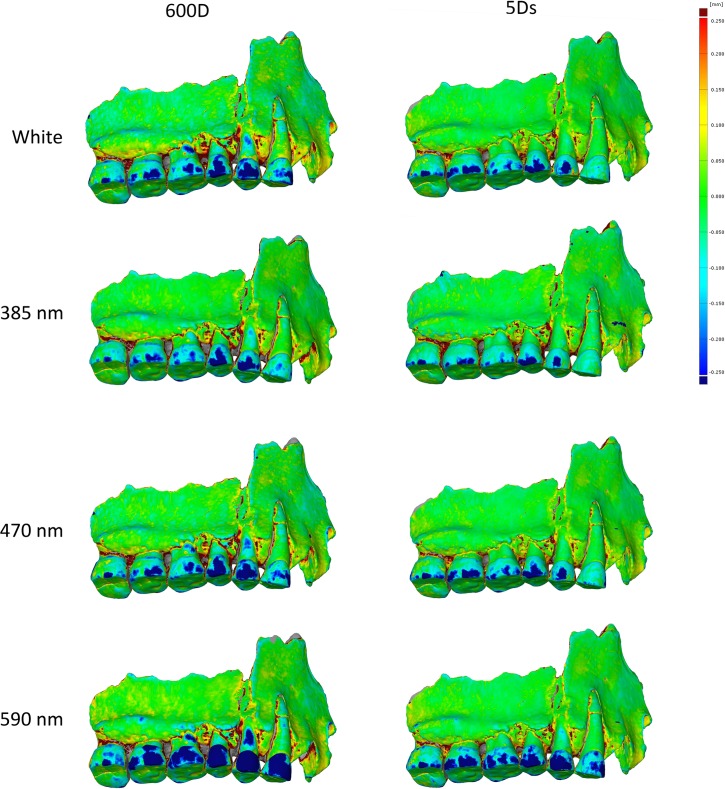
**Comparison between models captured with the Canon 600D (left) and the Canon 5Ds (right).** From top to bottom: white light, 385 nm, 470 nm and 590 nm.

We also compared the deviation scalar field between the models captured with the Canon DSLR 600D and 5Ds, processed for two rotations of pictures with the same parameters in Agisoft Photoscan. They showed that: (i) results of the processed 3D model from both DSLR showed less deviations in UV than in white; (ii) results from both camera display more deviations in longest wavelengths than in white; (iii) the models obtained with the 5Ds are a little more detailed than with the 600D, they also present a little less deviation. The 600D has a resolution of 18 Mpx while the 5Ds has a resolution of 50 Mpx: this explains why the models from the 5Ds are a little more detailed. But in general, the results are consistent between models obtained with the 600D and 5Ds (Fig 8).

**Fig 9:**
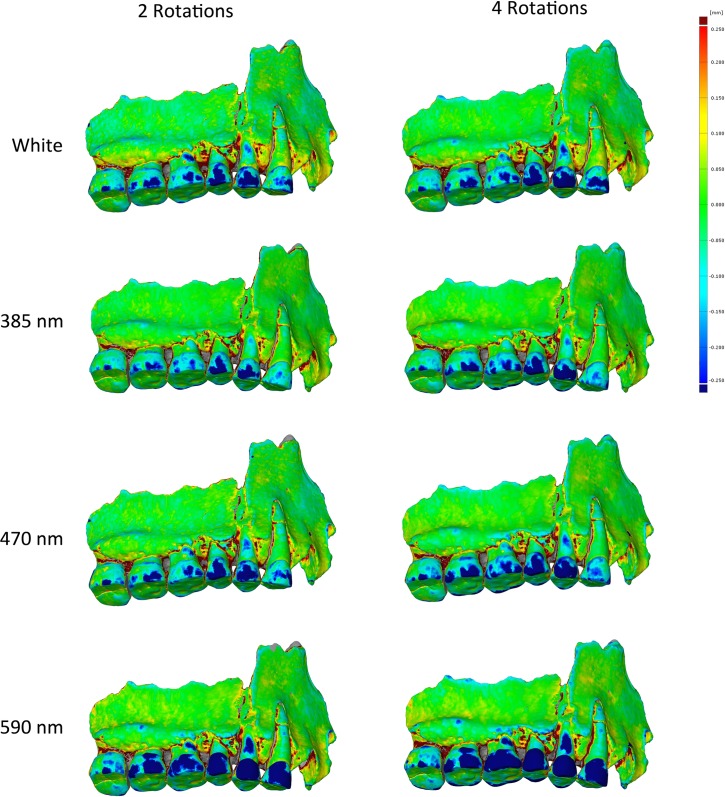
Models obtained with 2 rotations on the left, 4 rotations on the right (600D).

Comparison of the deviation scalar fields between models obtained between two and four rotations with the Canon 600D, processed with the same parameters, show that the extra two rotations increase a little the deviation areas on the enamel instead of reducing it (Fig 9).

**Fig 10:**
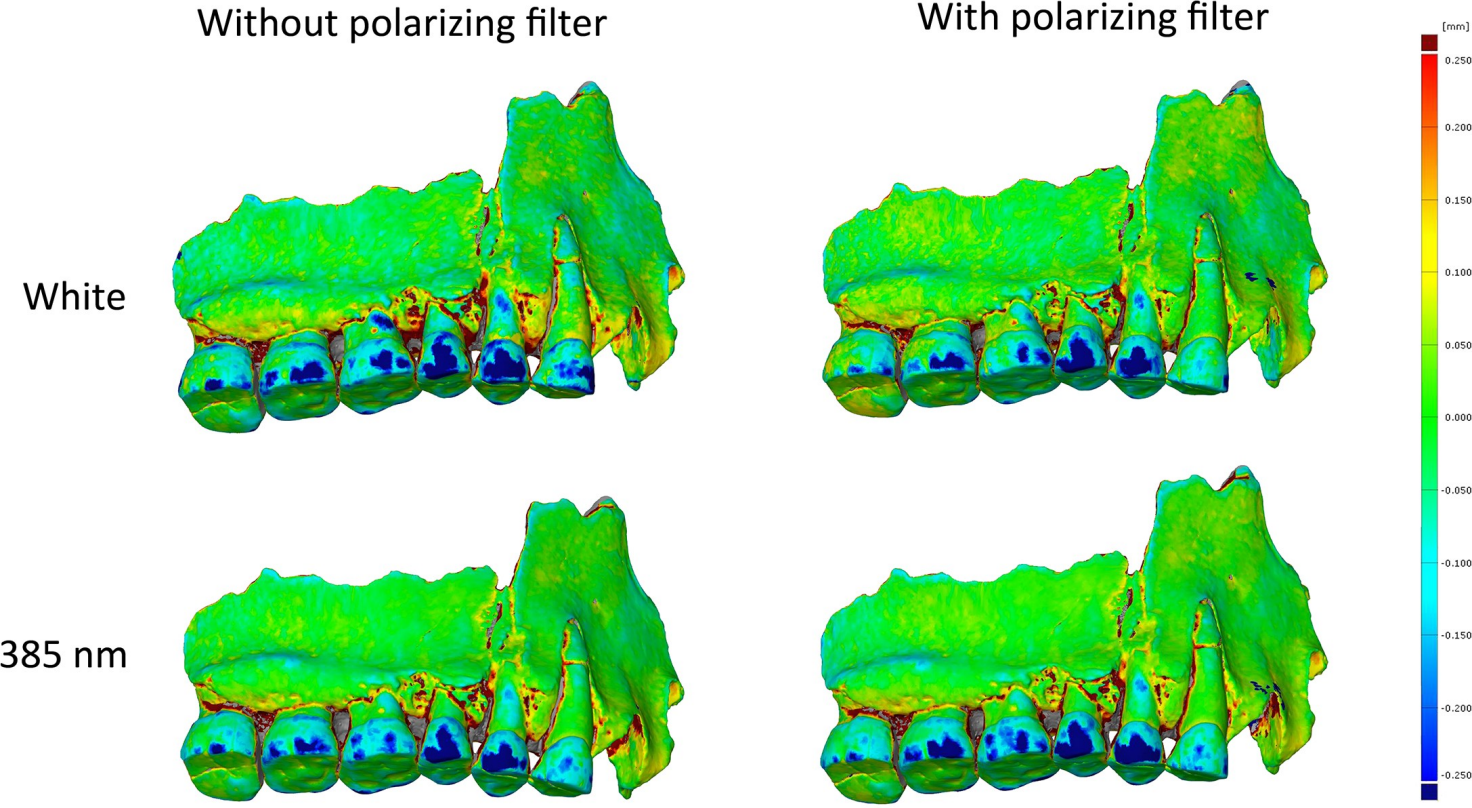
Comparison of Spy 2A (captured with the 600D) with and without a polarizing filter.

Spy 2A was also digitized with a circular polarizing filter for white light and 385 nm. The polarizing filter cut-off most of the UVR. The results with the polarizing filter improved the models in white light but the model was worse in UV according to the deviation area observed (Fig 10).

#### Expected accuracy

The photogrammetry models have a Ground Sampling Distance (GSD) of approximately 30 μm/pix. According to Vautherin *et al*. (2016) [44] the expected accuracy of a model is 2 to 3 times de GSD, therefore the accepted error of our models should be 90 μm. Besides GSD, camera settings, lens quality, sensor dynamic range, shooting process, overlap between images are among others also affecting the resolution of the photogrammetry model. As the resolution is unknown and given the absence of a CAD model, the accuracy measured is a relative accuracy to the μCT model.

**Fig 11:**
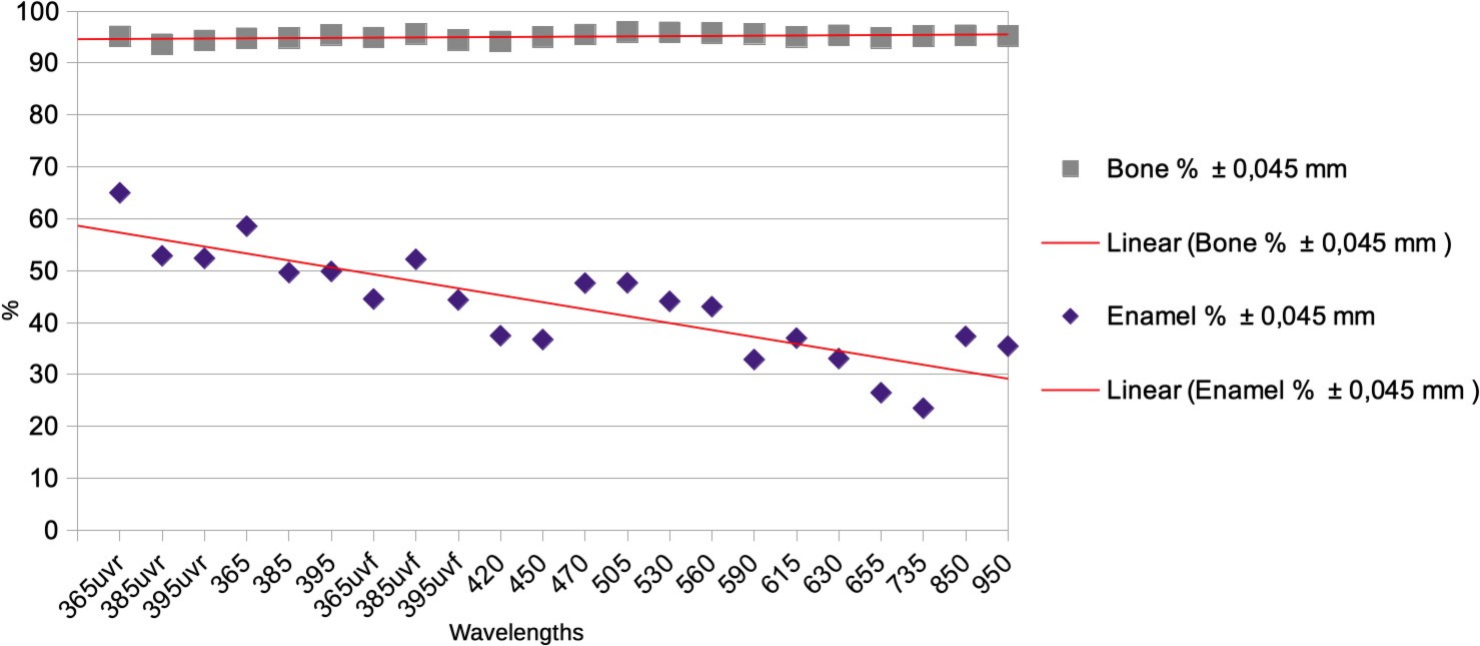
Graphic displaying the percentage of sampled point at +/- 45 μm of the reference model.

**Fig 12:**
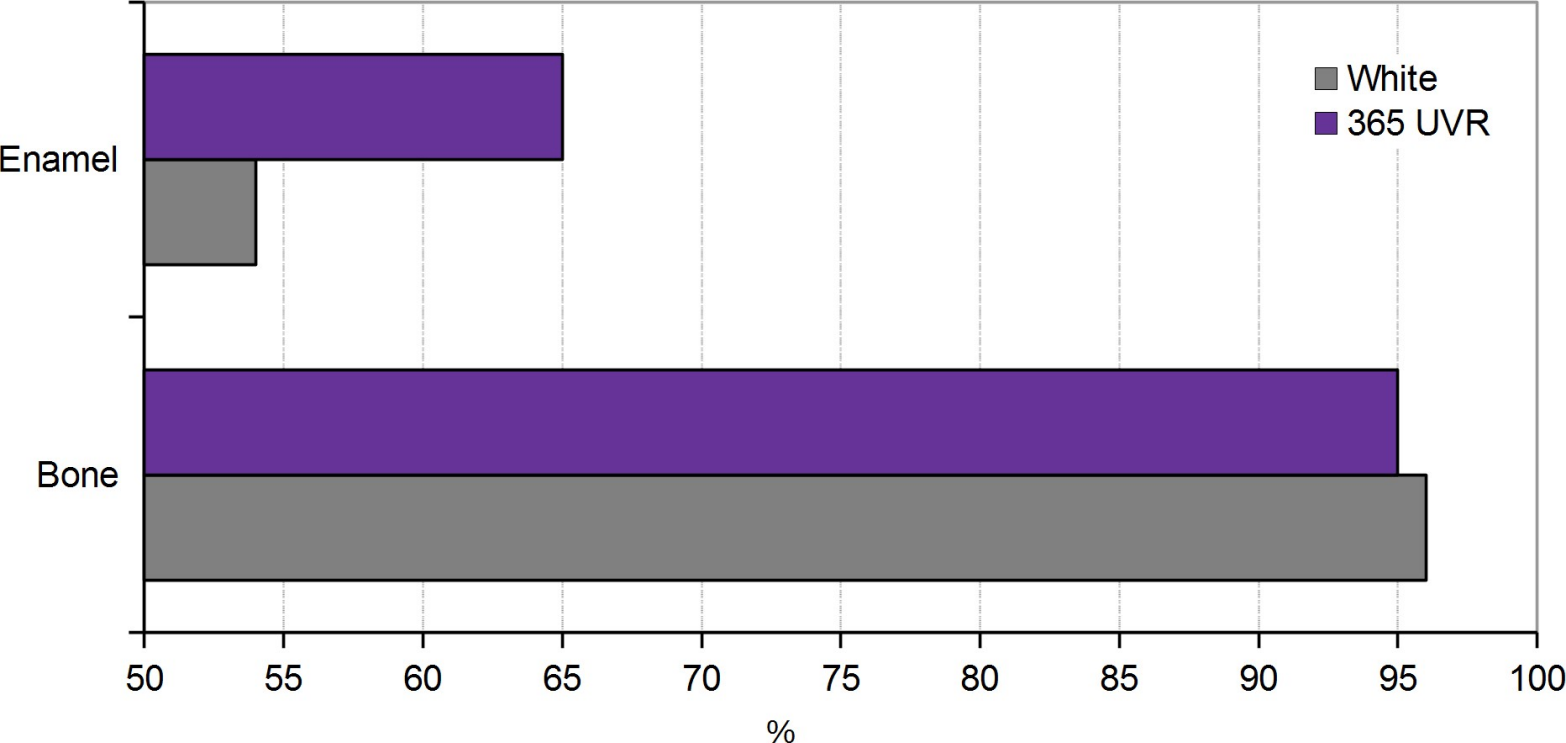
Graphic displaying the percentage of sampled point at +/- 45 μm of the reference model between white light and 365 nm UVR.

According to Table 2, for the bone part, in most of the wavelengths, 95% of the sampled points are in the acceptable error range of +/- 45 μm. For enamel, this error is much higher for all wavelengths: in the best cases (UVR365nm) 65% of the sampled points are in the +/- 45 μm range, in the worst cases (735 nm) only 23% of the sampled point are in the +/- 45 μm range. Although the percentages of accuracy of enamel are irregular, we can observe a general tendency of less accuracy when the wavelengths go toward 735 nm, while the best results are observed in UVR and UV (Fig 11). The model in white light is similar to 365 nm UVR for bone, but has more than 10% more errors for enamel (Fig 12).

**Table 2:** Sampled points at +/- 45 μm.

	Bone sampled points	Bone +/- 45 μm	Bone % +/- 45 μm	Enamel sampled points	Enamel +/- 45 μm	Enamel +/- 45 μm
**White**	8992	8625	**96**	13262	7103	**54**
**UVR 365**	9443	8979	**95**	9759	6340	**65**
**UVR 385**	9500	8890	**94**	10463	5528	**53**
**UVR 395**	8958	8447	**94**	9635	5044	**52**
**365**	10345	9801	**95**	11964	7004	**59**
**385**	10481	9937	**95**	11941	5922	**50**
**395**	9797	9345	**95**	12160	6059	**50**
**UVF 365**	7996	7584	**95**	15577	6934	**45**
**UVF 385**	6712	6414	**96**	11754	6130	**52**
**UVF 395**	8491	8014	**94**	11464	5081	**44**
**420**	8594	8089	**94**	10041	3760	**37**
**450**	9153	8694	**95**	11728	4305	**37**
**470**	8825	8422	**95**	11719	5575	**48**
**505**	8834	8476	**96**	12056	5742	**48**
**530**	8010	7679	**96**	12170	5362	**44**
**560**	8212	7863	**96**	12322	5302	**43**
**590**	8691	8309	**96**	16459	5407	**33**
**615**	7578	7201	**95**	16884	6242	**37**
**630**	10169	9692	**95**	21294	7035	**33**
**655**	7440	7056	**95**	15289	4045	**26**
**735**	8001	7611	**95**	16750	3934	**23**
**850**	10107	9633	**95**	19177	7156	**37**
**950**	10237	9742	**95**	18730	6637	**35**

Sampled points at +/- 45 μm to the total sampled points from a segment of bone and enamel. The number of sample points is automatically defined by CloudCompare.

#### Statistical analysis of the deviation

**Fig 13:**
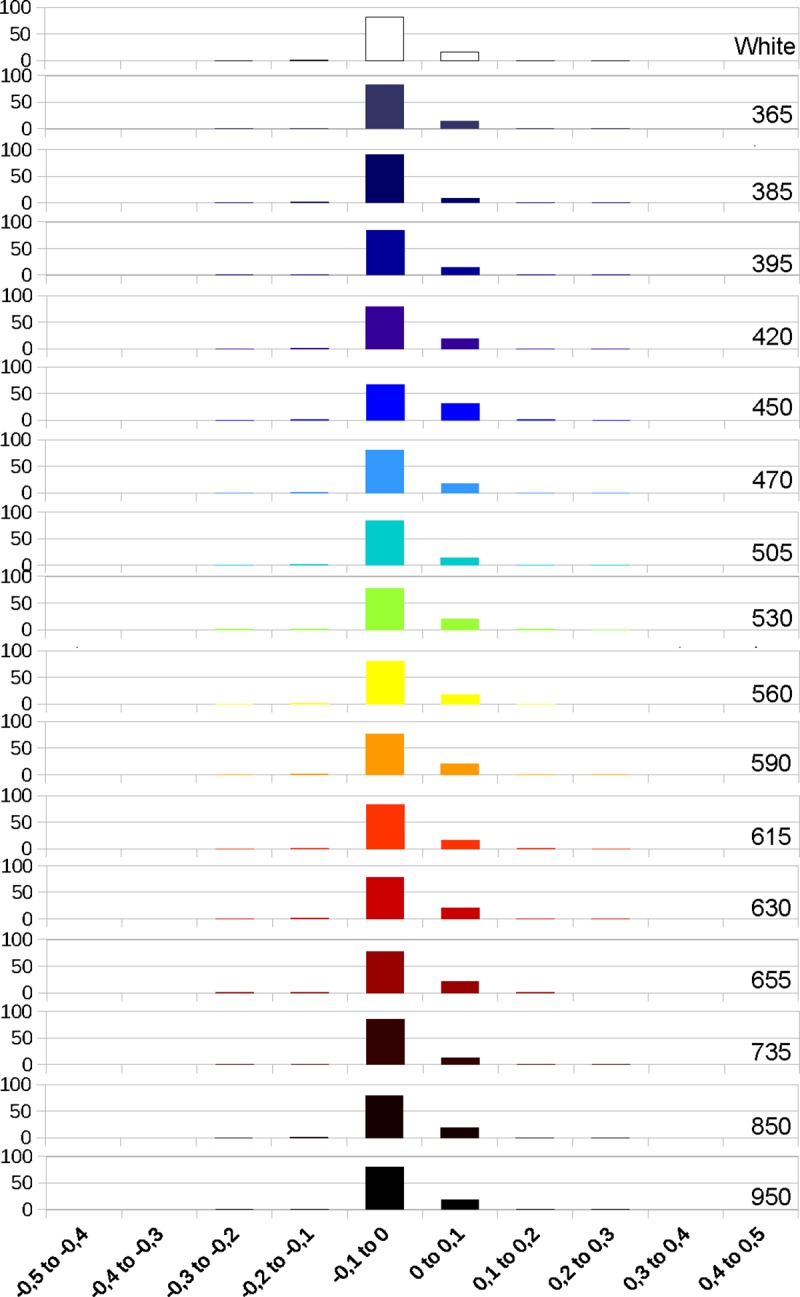
Graphic representing the deviation in percentages at different wavelengths on bone.

**Fig 14:**
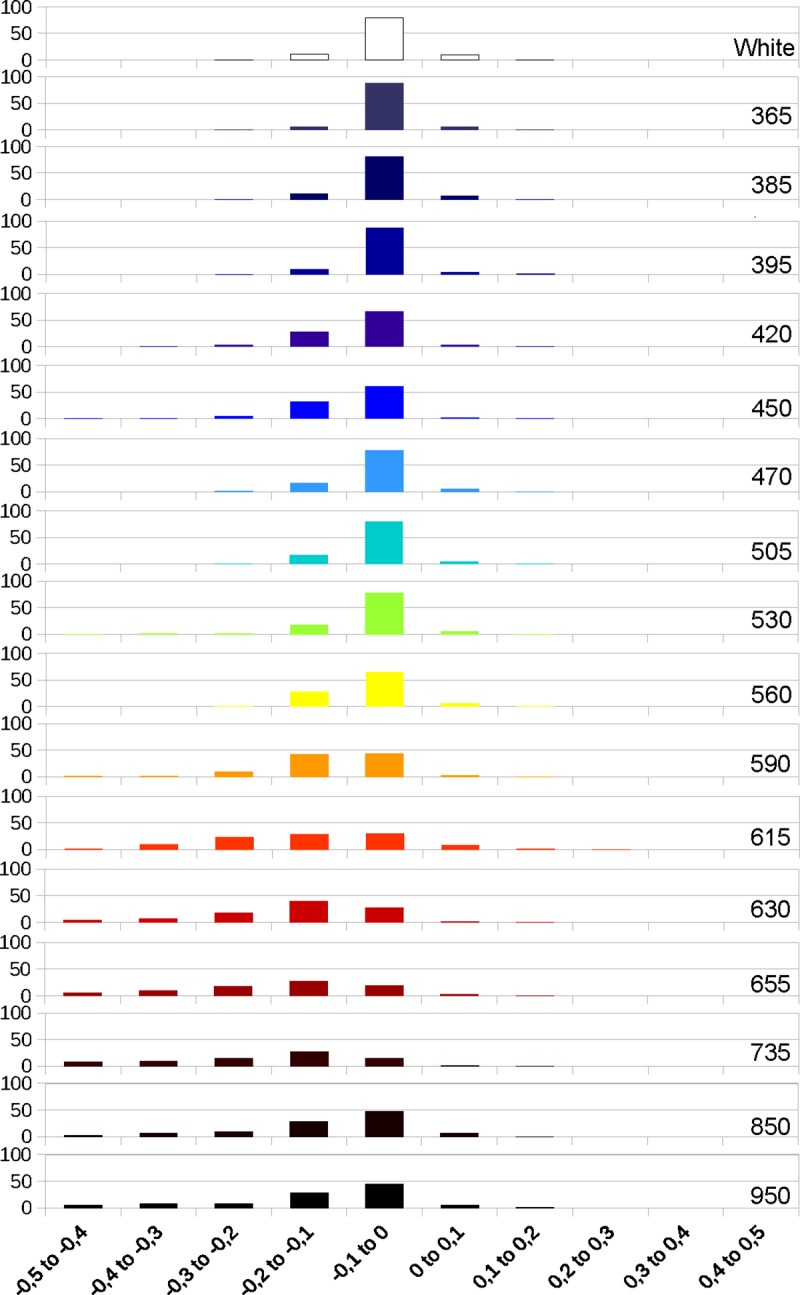
Graphic representing the deviation in percentages at different wavelengths on enamel (M1).

**Fig 15:**
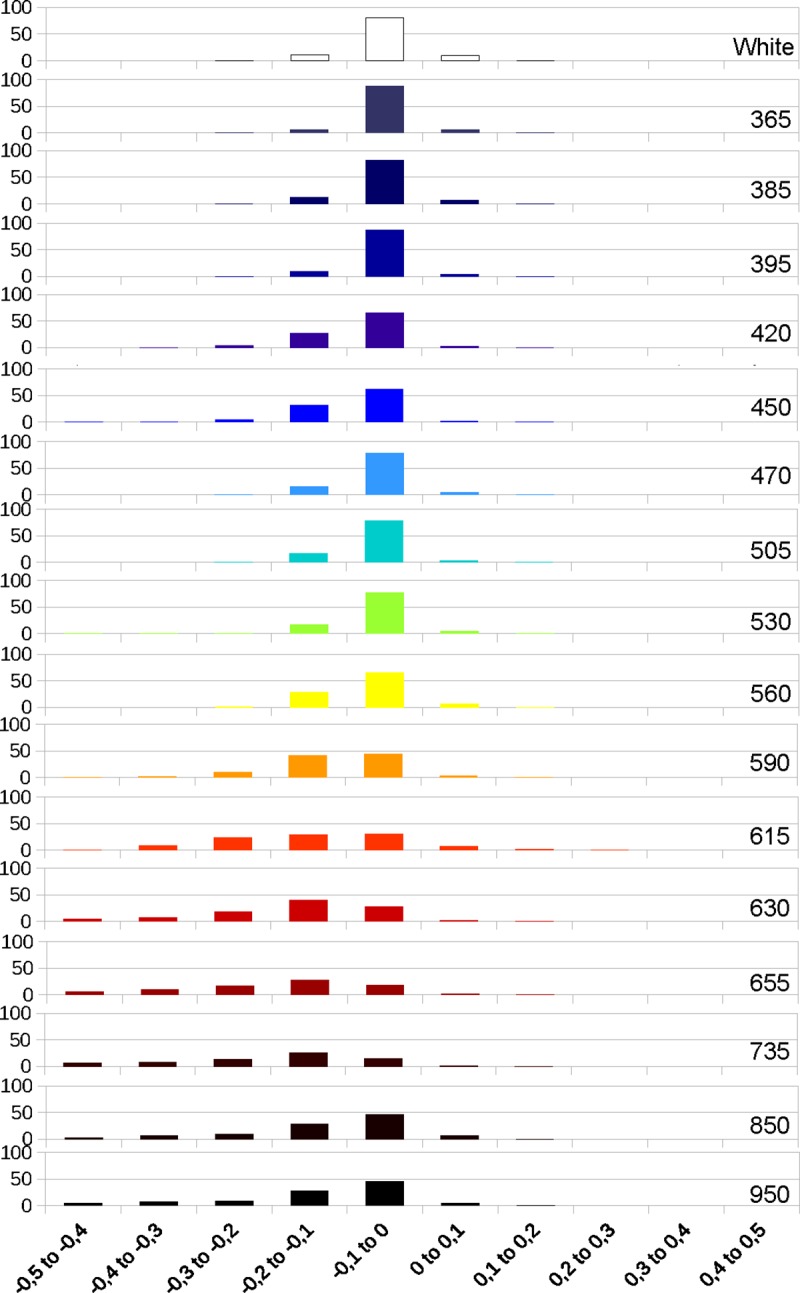
Graphic representing the deviation in percentages at different wavelengths on enamel (P1).

Next, we analyzed the deviations values between bone and enamel for the models obtained in photogrammetry at the different wavelengths. The graphics obtained from those values show that the deviations on the enamel get larger with the higher wavelengths, while bone remains relatively stable through the full spectrum (Figs 13, 14 and 15). The deviation on enamel is almost always negatives, this could be due to the translucency of the enamel. The deviation values at 850 and 950 nm are lower than for the longer wavelengths of the visible spectrum and 735 nm, but they are still higher than for white light. The graphics for enamel deviation (Figs 14 and 15) showed a shift of the major proportion of deviation values from the -0.1 to 0 category to -0.2 to -0.1 category for the wavelengths between 590 and 735 nm. The deviation for UV wavelengths is the least.

**Fig 16:**
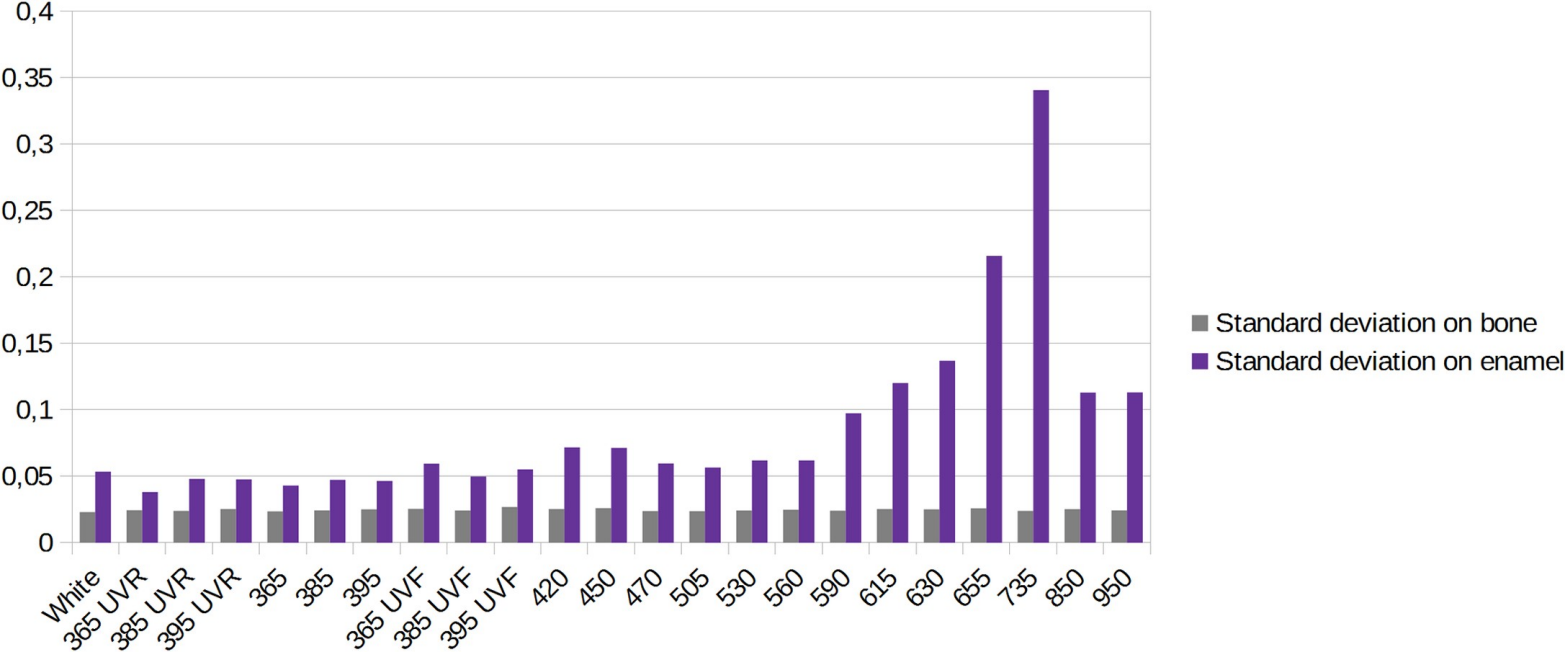
Graphic representing the standard deviation at different wavelengths for bone and enamel.

Subsequently, we compared the standard deviation between bone and enamel at the different wavelengths and again the standard deviation increases for enamel as the wavelengths get higher until 735 nm, then in IR at 850 nm and 950 nm it diminishes a little. The standard deviation is smaller in UV and UVR than in UVF and white lights models (Fig 16).

#### Qualitative analysis

**Fig 17:**
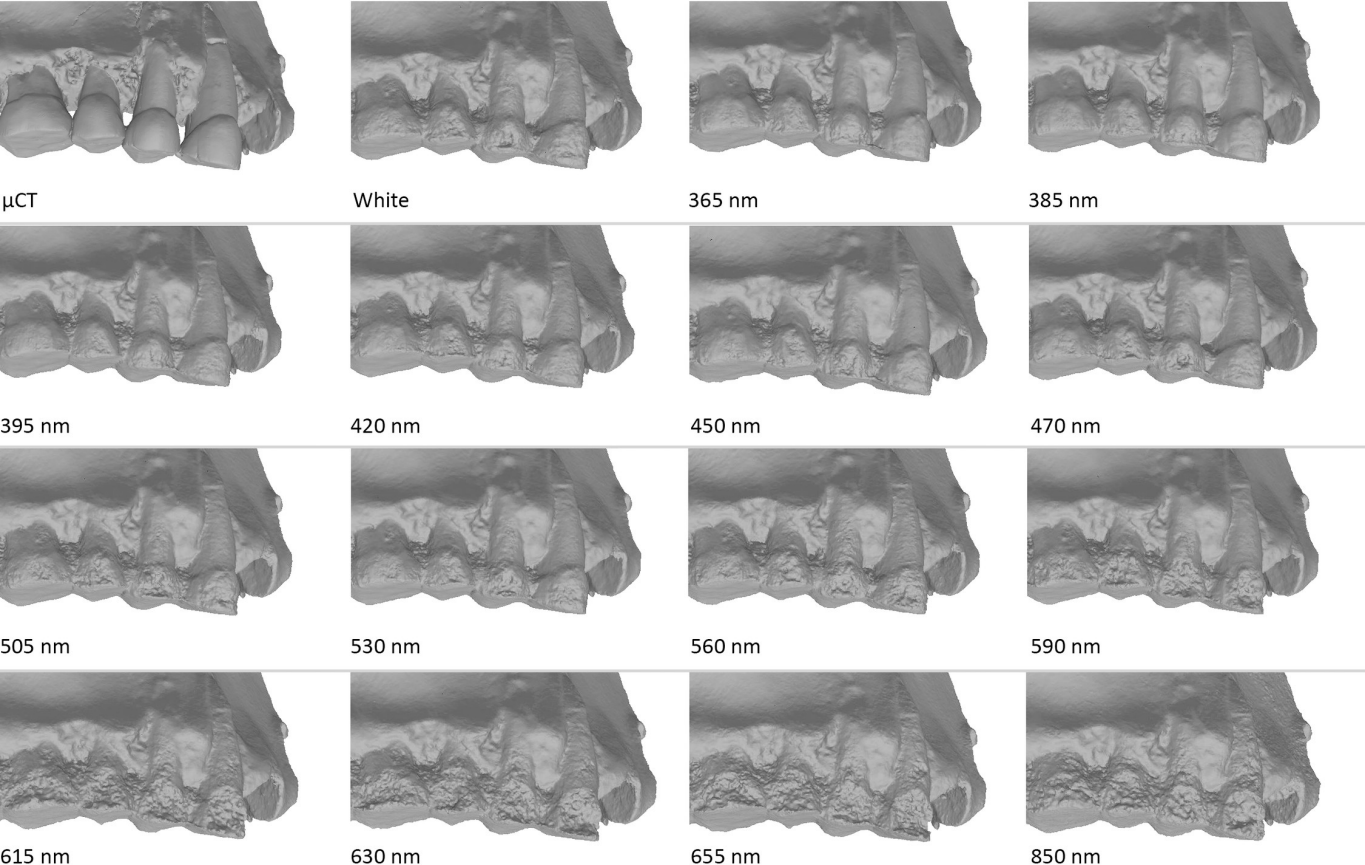
Spy 2A, models obtained with the 5Ds at the different wavelengths. First row (left to right): Picture in white light, White, 850 nm, 655 nm. Second row (left to right): 630 nm, 615 nm, 590 nm, 560 nm. Third row (left to right): 530 nm, 505 nm, 470 nm, 450 nm. Fourth row (left to right): 420 nm, 395 nm, 385 nm, 365 nm.

**Fig 18:**
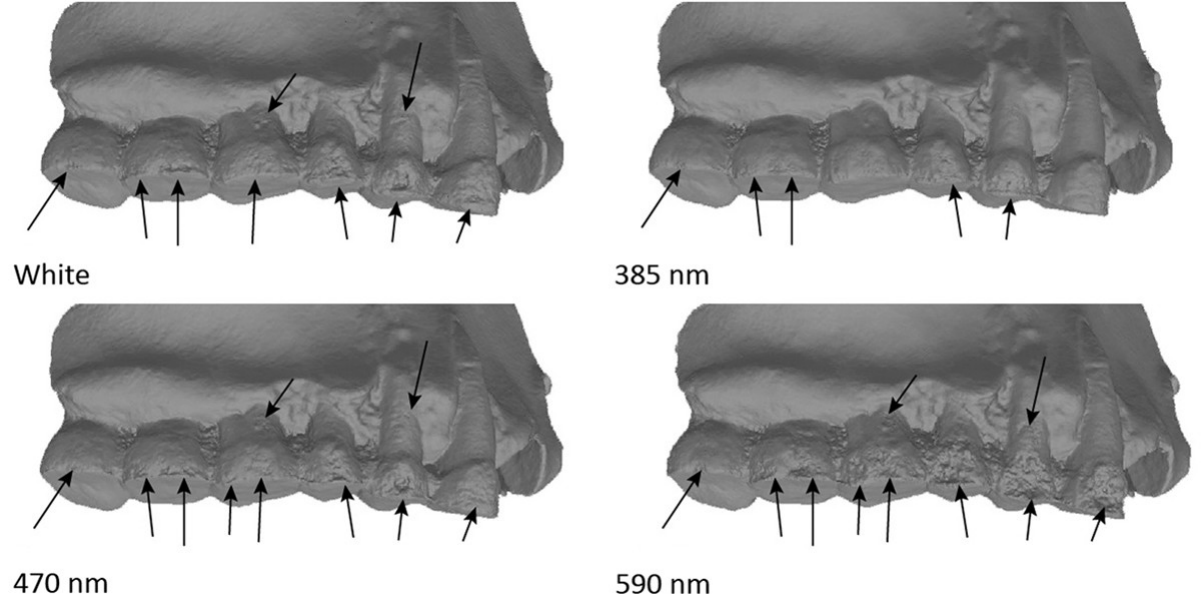
Details on the different models from the 5Ds captures of Spy 2A across 3 wavelengths and white light. Arrows indicate major artefacts or outliers.

**Fig 19:**
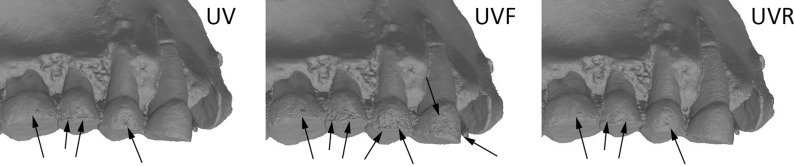
**Comparison between UV (left), UVF (middle) and UVR (right) at 365 nm (5Ds).** Arrows indicate major artefacts or outliers.

Finally, we confronted the deviation results from these analyses with what visually could be observed on the 3D models in confrontation to the real object. Models obtained in UV appear to be more accurate than in white light, but there are still some artefacts present on the 2 premolars (PM) and on the 2 last molars (M2 and M3), but these artefacts are smaller than with other wavelengths (Figs 17, 18 and 19). The models were carefully examined under a microscope and compared to a μCT model and the real teeth don’t present similar artefacts. The model in white lights is similar to the models obtained in the blue wavelengths (420–470 nm). The noise and outlier are much larger in the longer wavelengths.

Results from the different analysis, both from the deviation values and the qualitative analysis, concur to indicate that UV and specially UVR enable to improve the 3D model for the enamel parts.

### Validation of the photogrammetry results obtained with Spy 2A

As the qualitative evaluation correspond with what was observed in the statistical analysis of the deviations between models the rest of the analysis will be based only on qualitative analysis. The aim being to confirm or infirm what was observed with Spy 2A on the influence of the wavelengths and the enamel quality.

#### Dataset 2—Spy 2B (RBINS)

**Fig 20:**
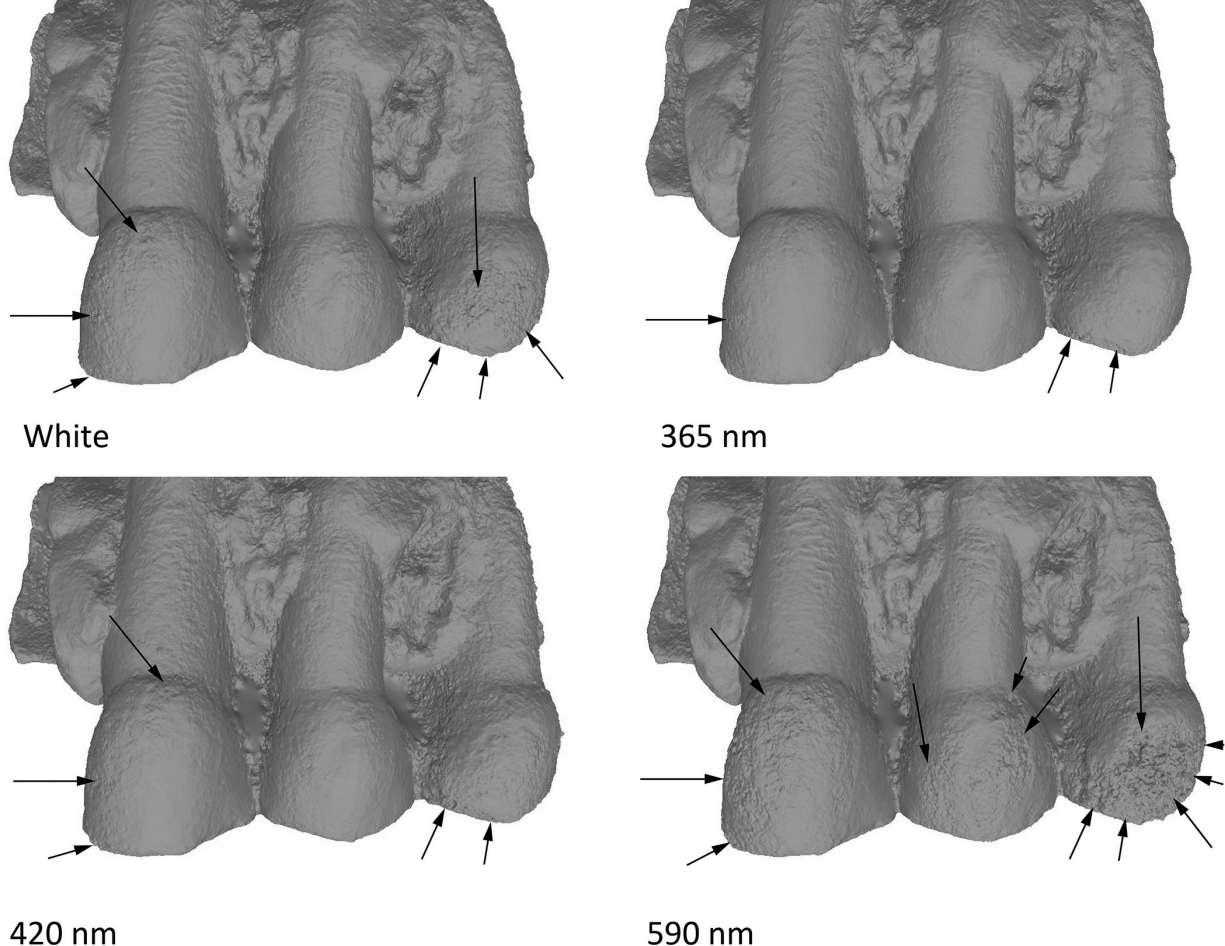
Spy 2B at different wavelengths. From top to bottom: White, 365 nm, 420 nm, 590 nm. Arrows indicate major artefacts or outliers.

Spy 2B was captured with the Canon 5Ds. Like for Spy 2A, results were best in UV (the best result is obtained at 365 nm) and models were worse in red (>590 nm) and IR wavelengths (735, 850, 950 nm). The model captured in white light is less good than in UV, but much better than in the longer wavelengths (Fig 20).

**Fig 21:**
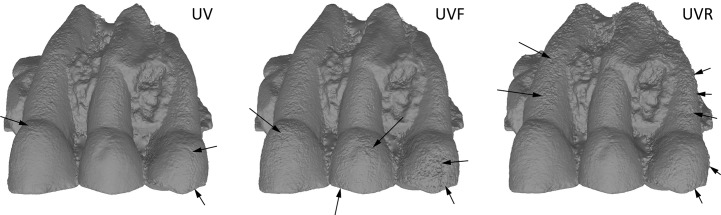
**Comparison between UV (left), UVF (center), UVR (right).** Arrows indicate major artefacts or outliers.

Comparison between models obtained in UV, UVF and UVR showed UVR results are better for enamel than in UVF (Fig 21). But the teeth root present more noise in UVR.

**Fig 22:**
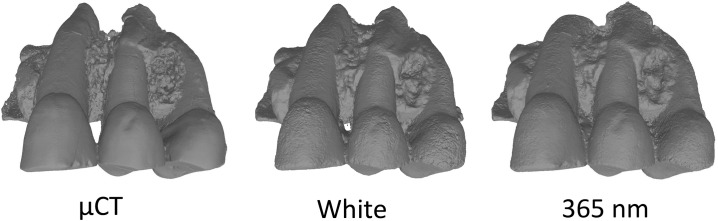
Comparison between photogrammetry at 365 nm and μCT models, non-smooth and smooth.

Models from multispectral photogrammetry (white and 365 nm) results were compared to a μCT model. The μCT model is smoother, but the general shape is the same. The μCT model displays less noise, sharper edges, more details for the cracks and doesn’t fill the gaps between the teeth (Fig 22).

#### Dataset 3—Koksijde (RBINS)

**Fig 23:**
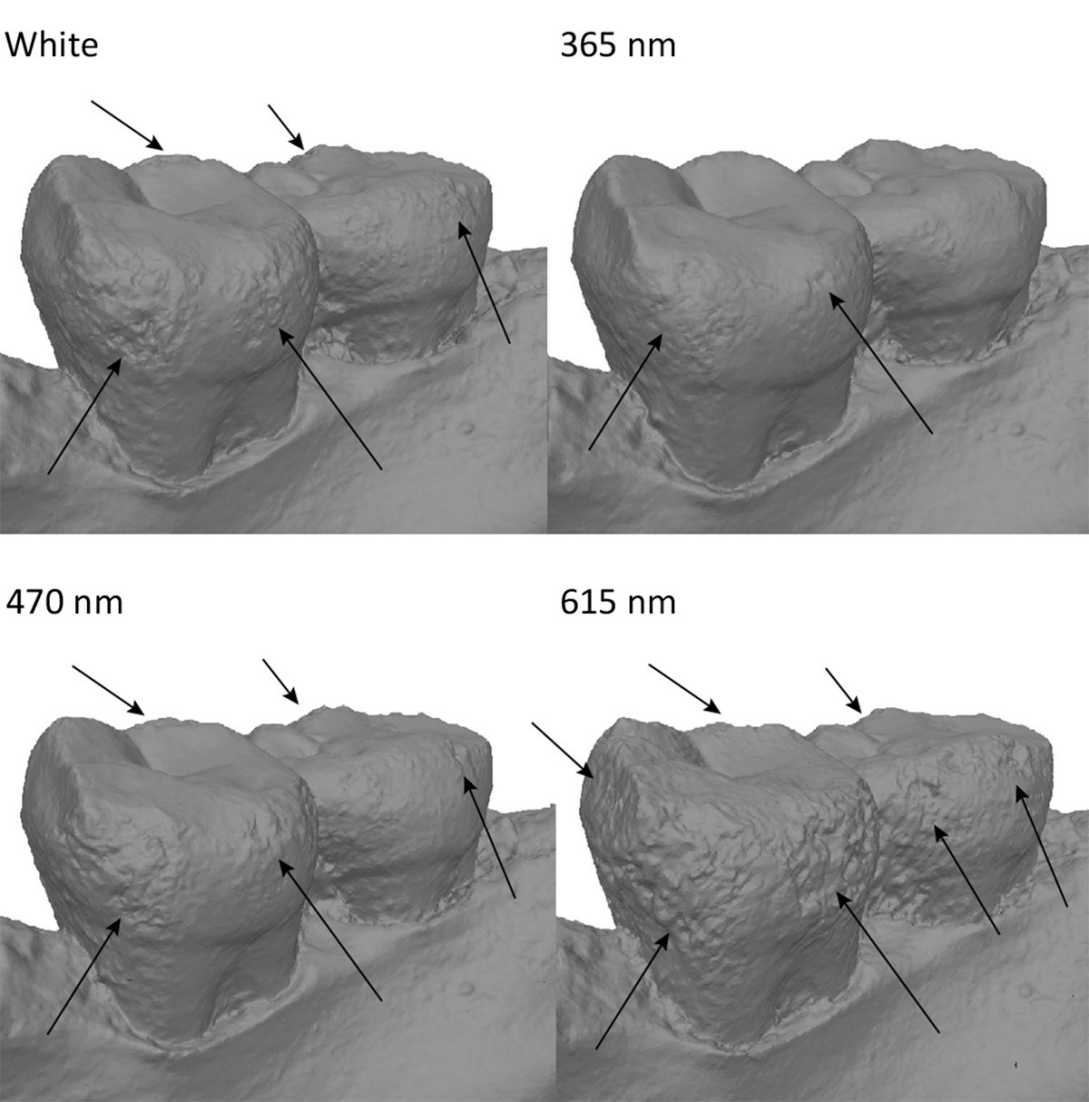
Molars from the mandible of Koksijde at different wavelengths. Arrows indicate major artefacts or outliers.

The teeth from this specimen are less reflective than the ones from Spy. They were captured only by the 5Ds DSLR. When observing the two molars of the Koksijde mandible, the best result was obtained at 365 nm, whilst the models in the reddish (>590 nm) and infrared wavelength were the least accurate. The blue model (420 nm in Fig 23) is more or less equivalent in quality to the white model. No difference is observed on the bone structure of the mandible.

#### Dataset 4—*Panthera leo* (R.G.11661, RMCA)

**Fig 24:**
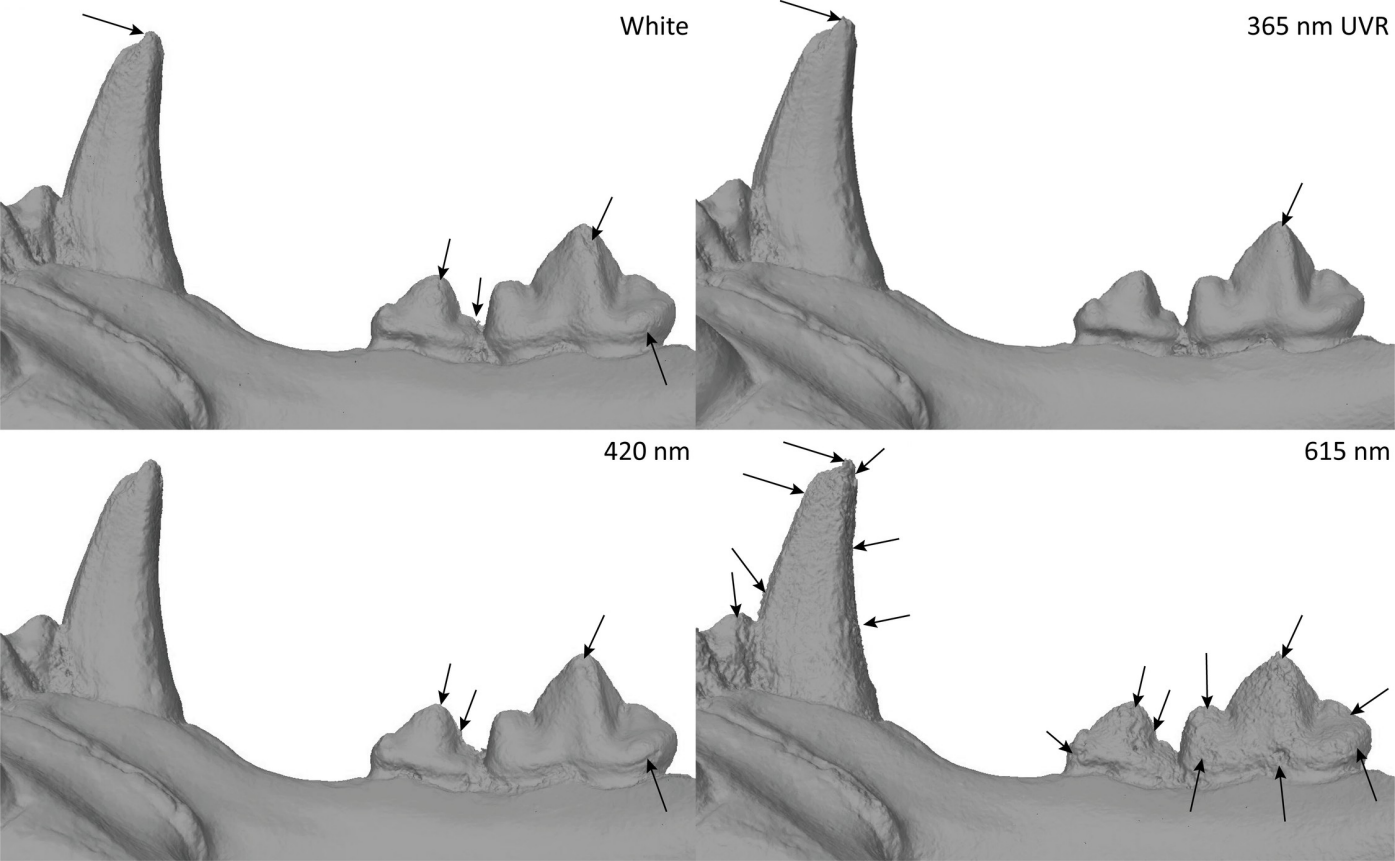
Details of *Panthera leo* teeth in white light, 365 nm UVR, 420 nm and 615 nm. Arrows indicate major artefacts or outliers.

The lion mandible was captured with the Canon 5Ds. The best result was obtained at 365 nm UVR. As for the previous results: the wavelengths in the green, red and IR were less accurate than the models obtained in white light and with UV/UVR wavelengths (Fig 24).

#### Dataset 5—*Panthera pardus* (R.G.35151, RMCA)

**Fig 25:**
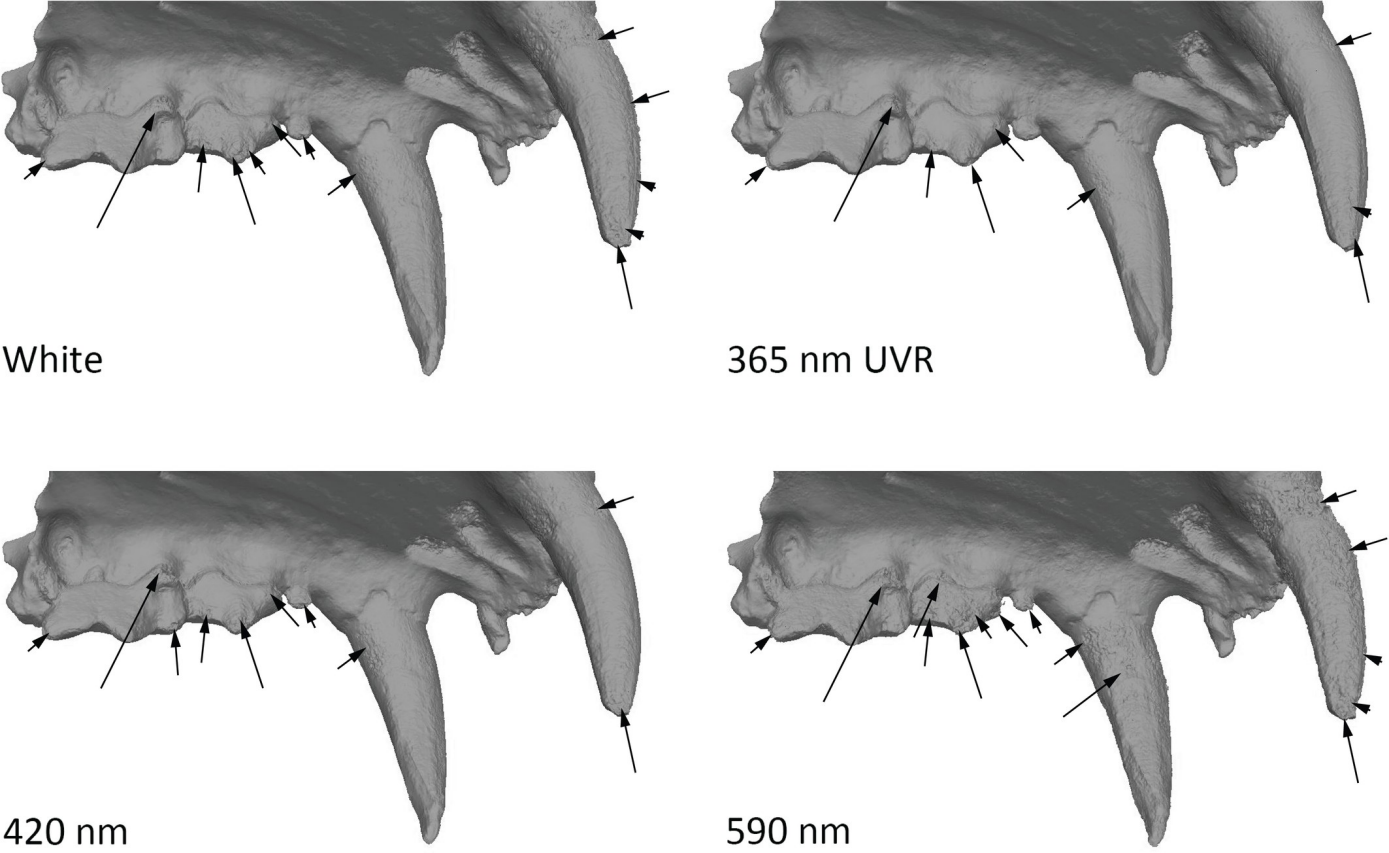
Differences between models of *Panthera pardus* at different wavelengths. Arrows indicate major artefacts or outliers.

The results shows photogrammetry with UV produces the best models. Blue light (420–470 nm) model is also a bit better than the white light model. Green light (505–530 nm) model is a little worse than the white light model. From the orange light (>590 nm) model onwards the enamel is badly rendered (Fig 25). The bone is similarly captured with most of the wavelengths.

**Fig 26:**
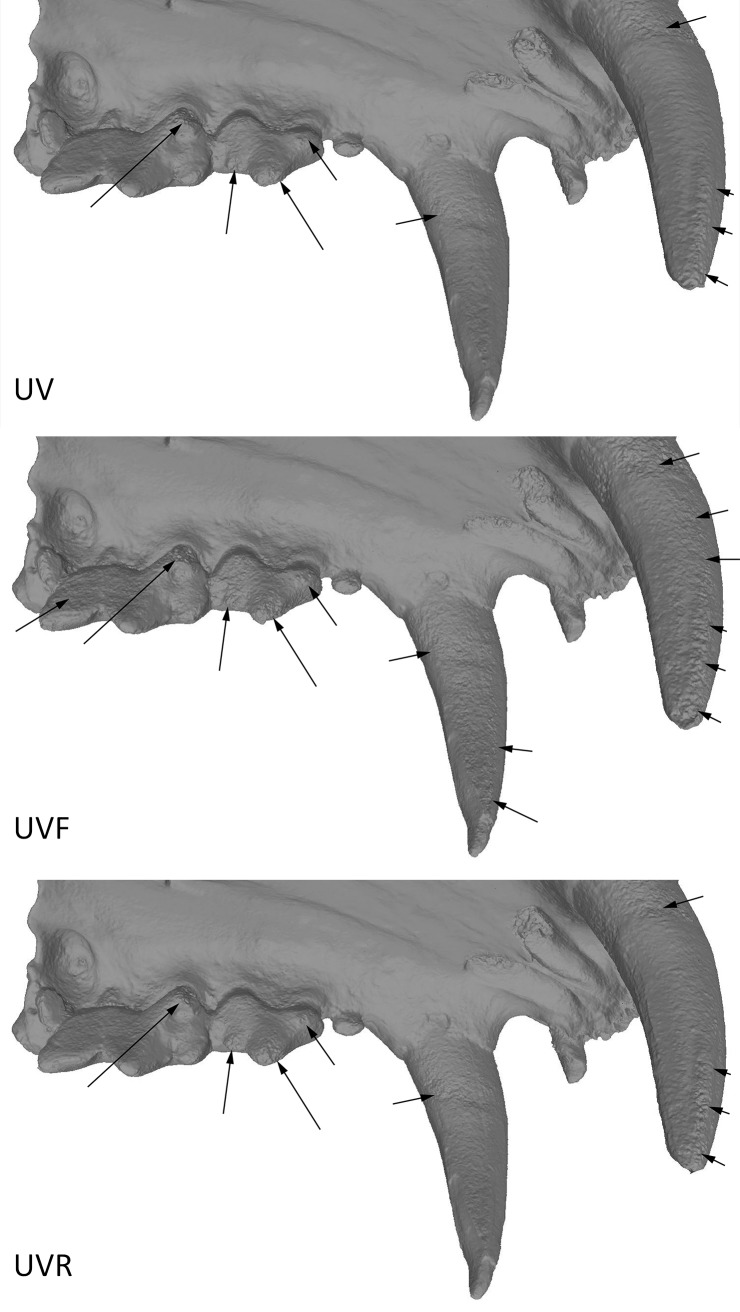
Differences between models of *Panthera pardus* in UV, UVF and UVR. Arrows indicate major artefacts or outliers.

Regarding UV: models in full UV and UVR are very similar, and are better than models in UVF (Fig 26).

**Fig 27:**
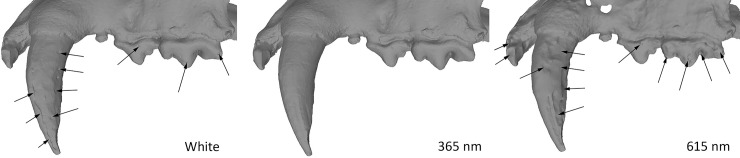
Models of *Panthera pardus* reconstructed in Bentley context capture software in white light, UV and red light. Arrows indicate major artefacts or outliers.

As for the others specimens, the quality of the models is better at certain wavelengths. The question of the responsibility of the processing software has to be raised. Therefore a few of the models were reprocessed with Context Capture from Bentley, but the results obtained presented similar issues for the enamel (Fig 27), discarding software related issues.

#### Dataset 6—*Hyaena* sp (R.G.12814, RMCA)

**Fig 28:**
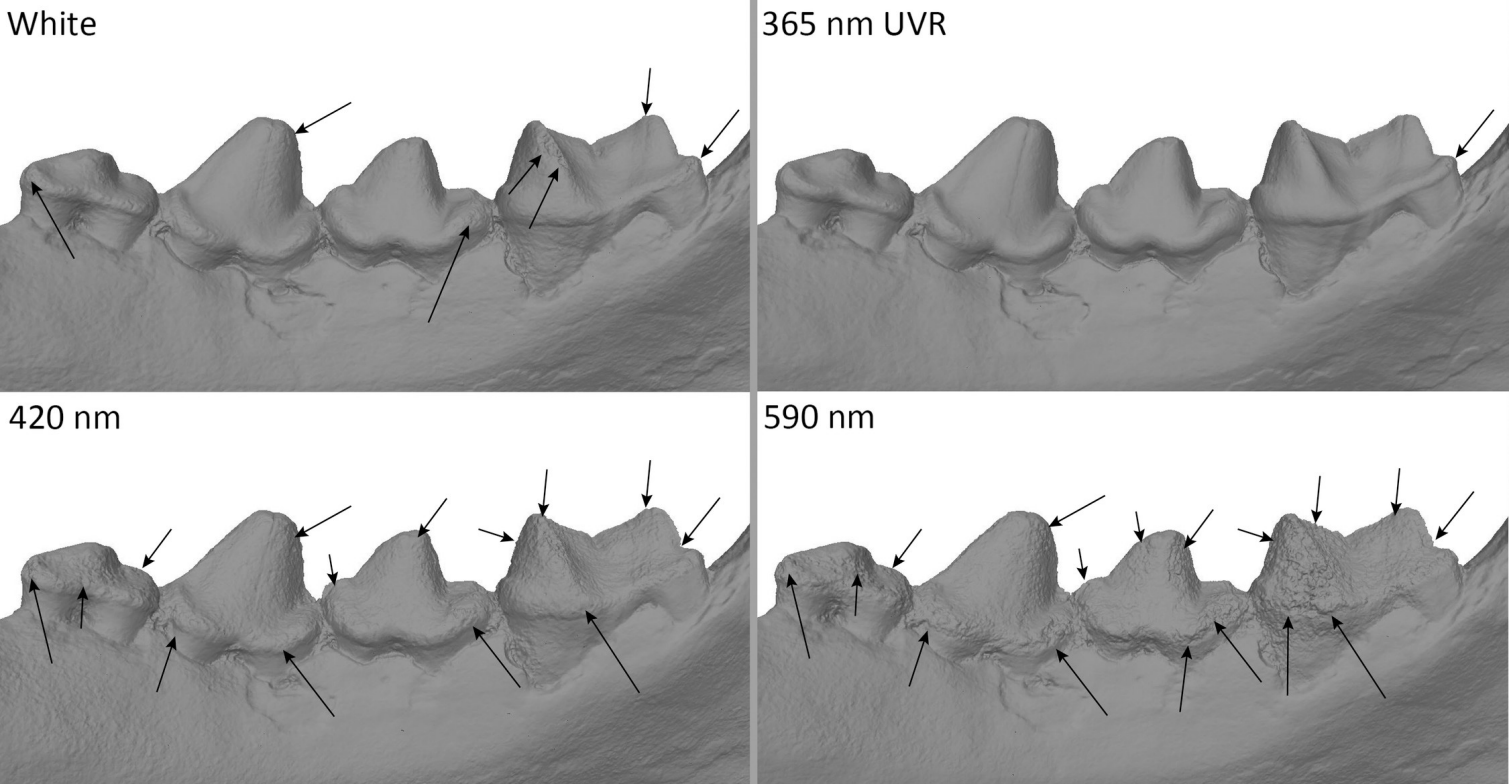
Differences between models at different wavelengths. Arrows indicate major artefacts or outliers.

**Fig 29:**
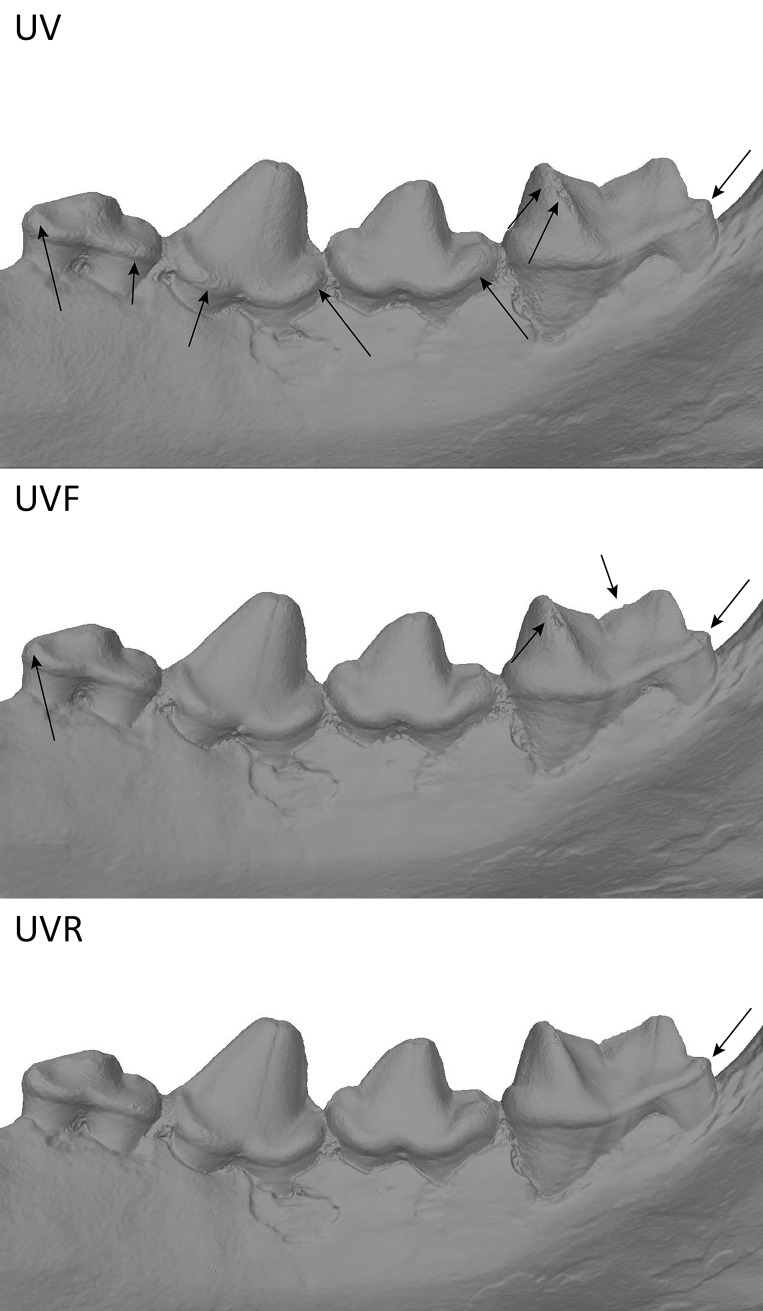
Differences between models in UV, UVF and UVR. Arrows indicate major artefacts or outliers.

**Fig 30:**
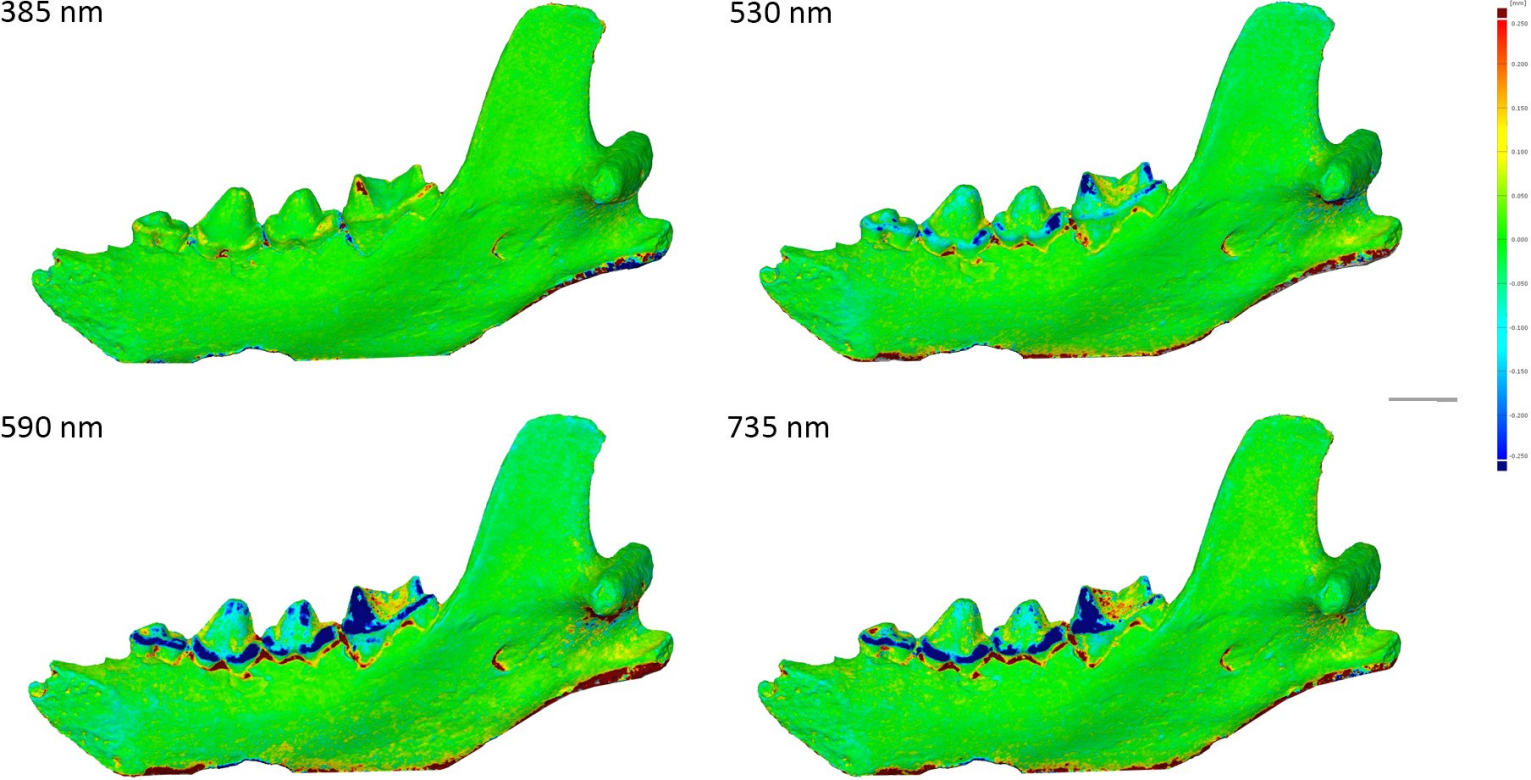
Scalar field representing the surface deviation in GOM Inspect displaying the differences between the white light model and, from left to right and from top to bottom, 385 nm, 530 nm, 590 nm and 735 nm.

Models of the Hyena mandible captured with the 5Ds. Results showed that in green wavelengths and above models are less detailed for enamel than what can be observe in UV and in white light (Fig 28). In general, green, yellow, red, IR are less accurate than the white light model, blue and UV models. The comparison between UVF, UVR and UV shows that UVR has less noise than the other two and in this case UVF is better than UV (Fig 29). The amount of details observed on the bone structure remains the same through the complete spectrum (Fig 30, the bottom part wasn’t captured, explaining the differences in the bottom part).

## Discussion

The comparison of Spy 2A between the different photogrammetry models and other digitization techniques showed that UVR photogrammetry is a good alternative for digitization of enamel.

The six case studies showed that there are differences between the final photogrammetry models when using different wavelengths. Those differences appear to be limited mainly to the enamel surface, but doesn’t seem to be affecting bone structure: the reconstruction of the bone surface is of a similar quality in all the wavelengths (95% of the points at less than +/- 45 μm). In general, the surface models of the enamel parts present less deviation, noise and outliers in the UV wavelengths (both UV and UVR). According to qualitative analyses, the white light models were of more or less equivalent quality to the models captured in the blue wavelengths and better in term of amount of deviation and standard deviation. The models in the longer wavelengths (yellow, orange, red, IR) displayed a large quantity of noise and outliers on the surface of the enamel. They are considered as inaccurate to white light and UV wavelengths models. The geometric surface quality of the models decreases as the wavelengths get longer.

UV, UVF and UVR were compared and in most cases UVR produced the models with less noise or outliers, but the difference between UV and UVR is very low. In UV and UVR, the best results are obtained in the lower wavelengths (365 nm). UVF produced results with more deviation and noise than the two others, as can be expected as it captures only emitted visible light (around 450nm [45–46]) instead of reflected UV. Therefore UVF can be considered as blue wavelengths and result showed that indeed the amount of error between UVF and blue is similar.

Models between the modified Canon 600D and the modified Canon 5Ds had similar problems in terms of noise and outliers on the enamel, but the models from the 5Ds have a bit more detail because of the greater resolution of the images captured with the 5Ds.

Having two or four rotations in the camera network for 3D photogrammetric reconstruction had very little impact on the quality of the enamel, but there was less noise on the bone surfaces.

**Fig 31:**
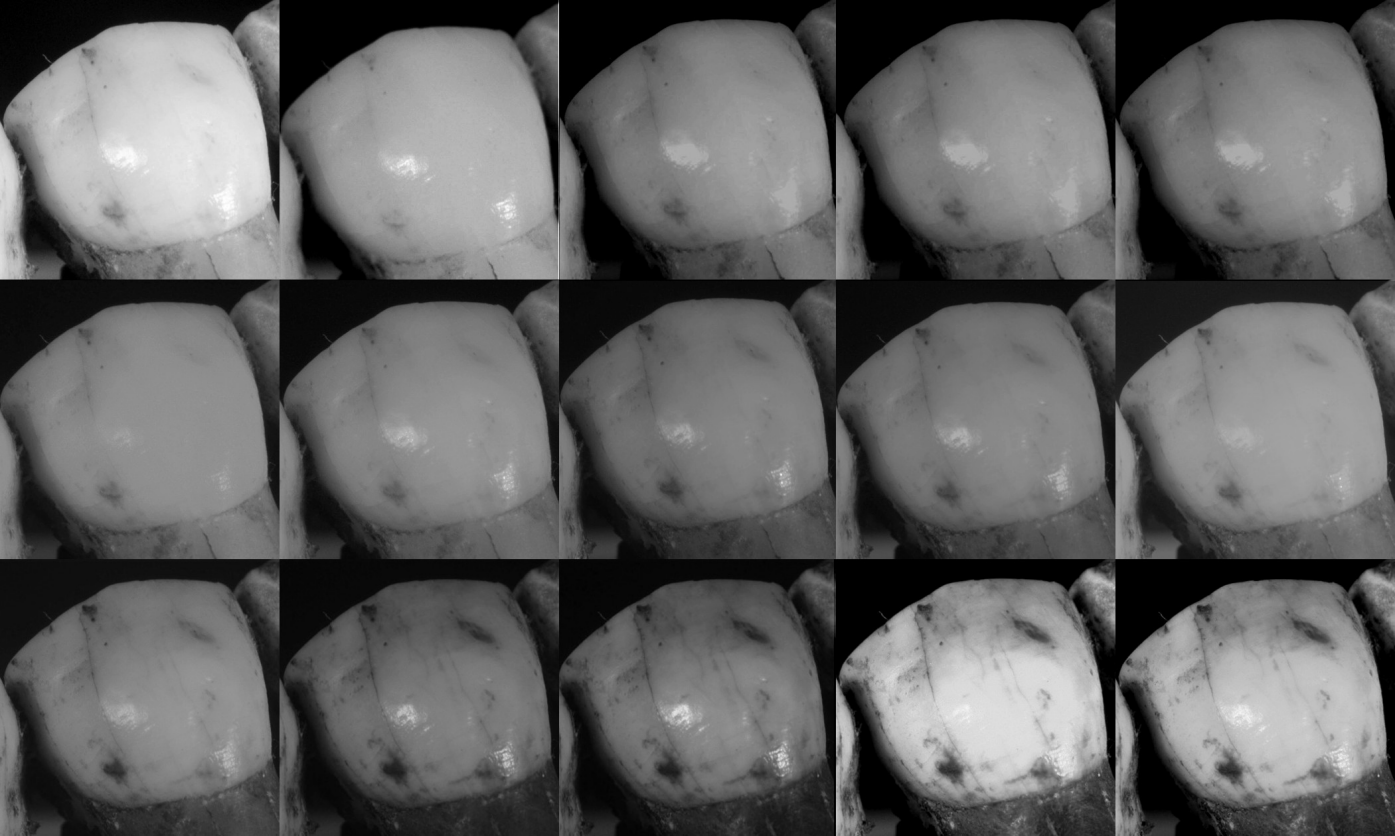
Detailed image of the enamel from Spy 2B at different wavelengths. Converted to grayscale to remove the risk of misinterpretation due to colors. First row (left to right): White, 850 nm, 655 nm, 630 nm, 615 nm. Second row (left to right): 590 nm, 560 nm, 530 nm, 505 nm. Third row (left to right): 470 nm, 450 nm, 420 nm, 395 nm, 385 nm, 365 nm.

**Fig 32:**
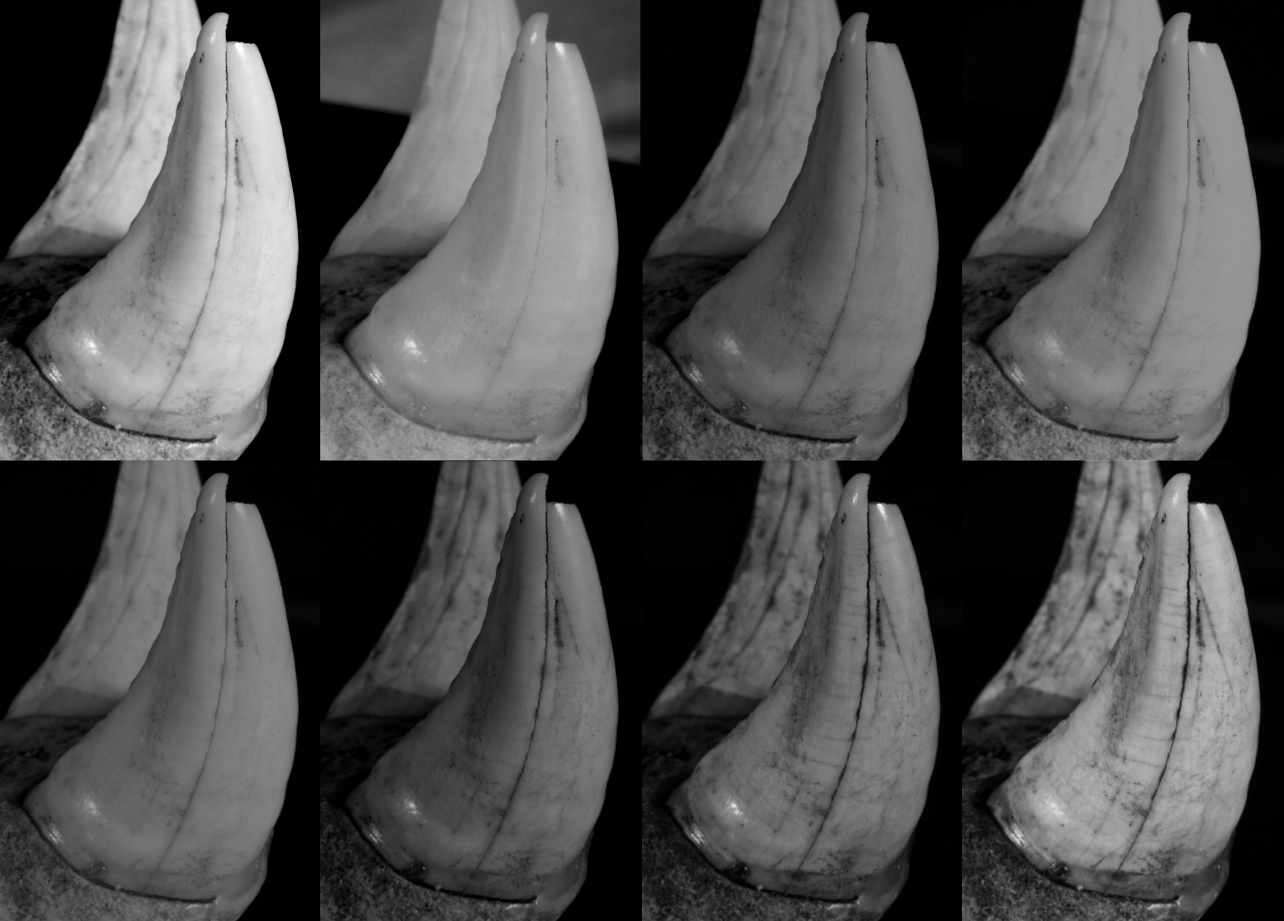
Detailed image of the enamel from the *Panthera leo* at different wavelengths. Converted to grayscale to remove the risk of misinterpretation due to colors. First row (left to right): White, 850 nm, 630 nm, 590 nm. Second row (left to right): 560 nm, 505 nm, 420 nm, 365 nm.

**Fig 33:**
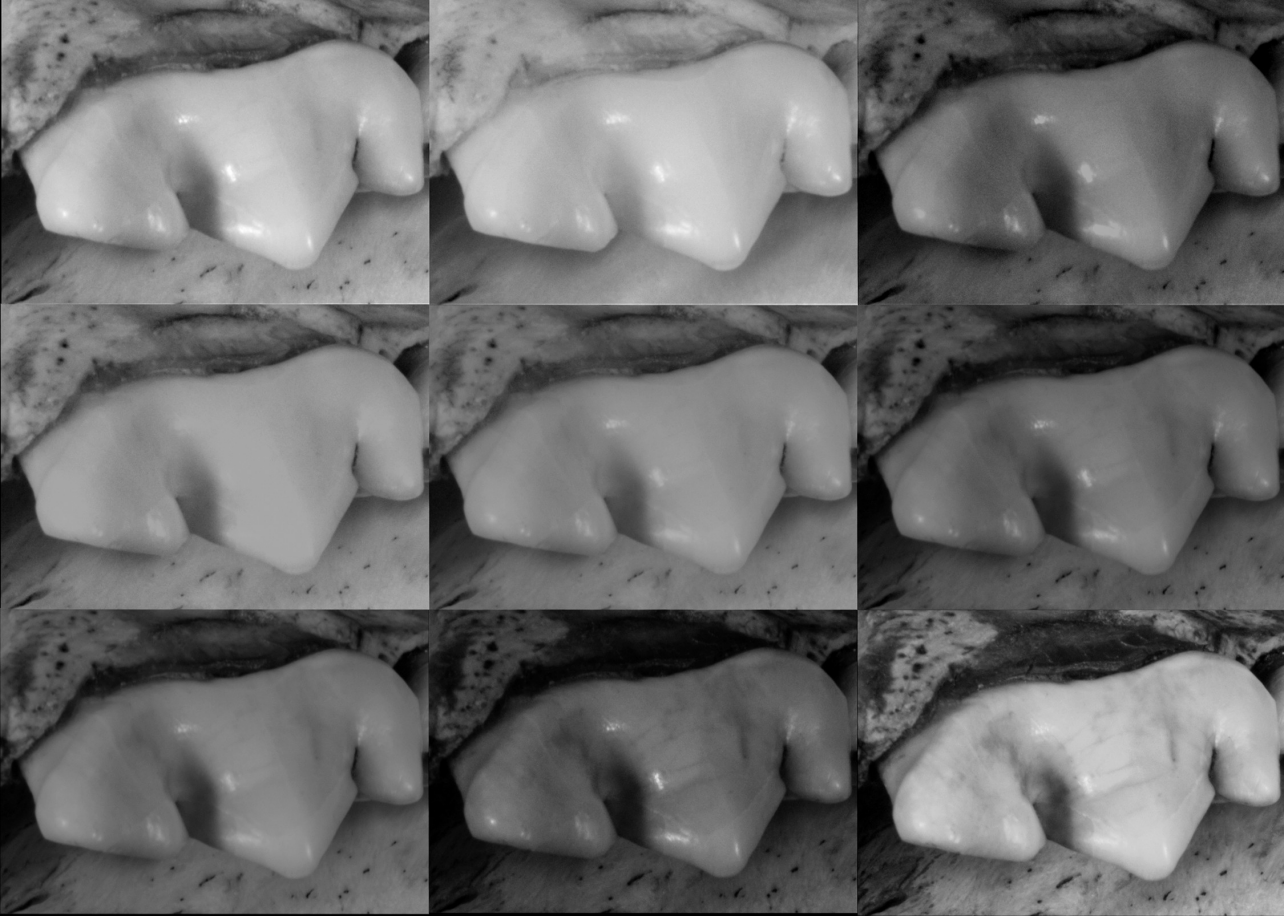
Detailed image of the enamel from the *Panthera pardus* at different wavelengths. Converted to grayscale to remove the risk of misinterpretation due to colors. First row (left to right): White, 850 nm, 630 nm. Second row (left to right): 590 nm, 560 nm, 505 nm. Third row (left to right): 470 nm, 420 nm, 365 nm.

The detailed pictures of the enamel were then analyzed. Results showed that the pictures in UV have more contrast and more details than the pictures in red or infrared (Figs 31, 32 and 33). If there are fewer details on the picture, it is normal that the photogrammetry models are less accurate because, as mentioned previously, it is difficult to perform photogrammetry on objects with featureless surfaces. The absence of detail on the pictures captured with longer wavelengths can be explained by the optical properties of the enamel. Light (UVA, visible light and NIR) is part of the electromagnetic spectrum. When an electromagnetic radiation reaches a surface or a medium, part of it is absorbed, part is reflected or scattered and a last part is further transmitted. These parts vary according to the nature of the material and the wavelengths. This means that depending on the material some wavelengths can travel (penetrate) far into the material or may be absorbed very quickly.

The main mineral component of both bone and enamel is calcium phosphates under the form of hydroxyapatite. Although their composition is quite similar the crystallinity index and the crystalline size of bone and enamel is very different, explaining their different optical properties [45, 47]. Enamel studies show that absorption is very weak in the visible range and increases in UV. Studies also showed enamel translucency increases with the wavelengths up to 525 nm [32]. Yellow and red wavelengths are highly transmitted by enamel [45]. Dentin and bone have a higher absorption coefficient (e.g. less transmissive) in the visible range than enamel [48]. This explains why there are less details on the pictures of the enamel in the yellow and red wavelengths and why bone and cementum are well represented through all the wavelengths.

## Conclusions & perspectives

**Fig 34:**
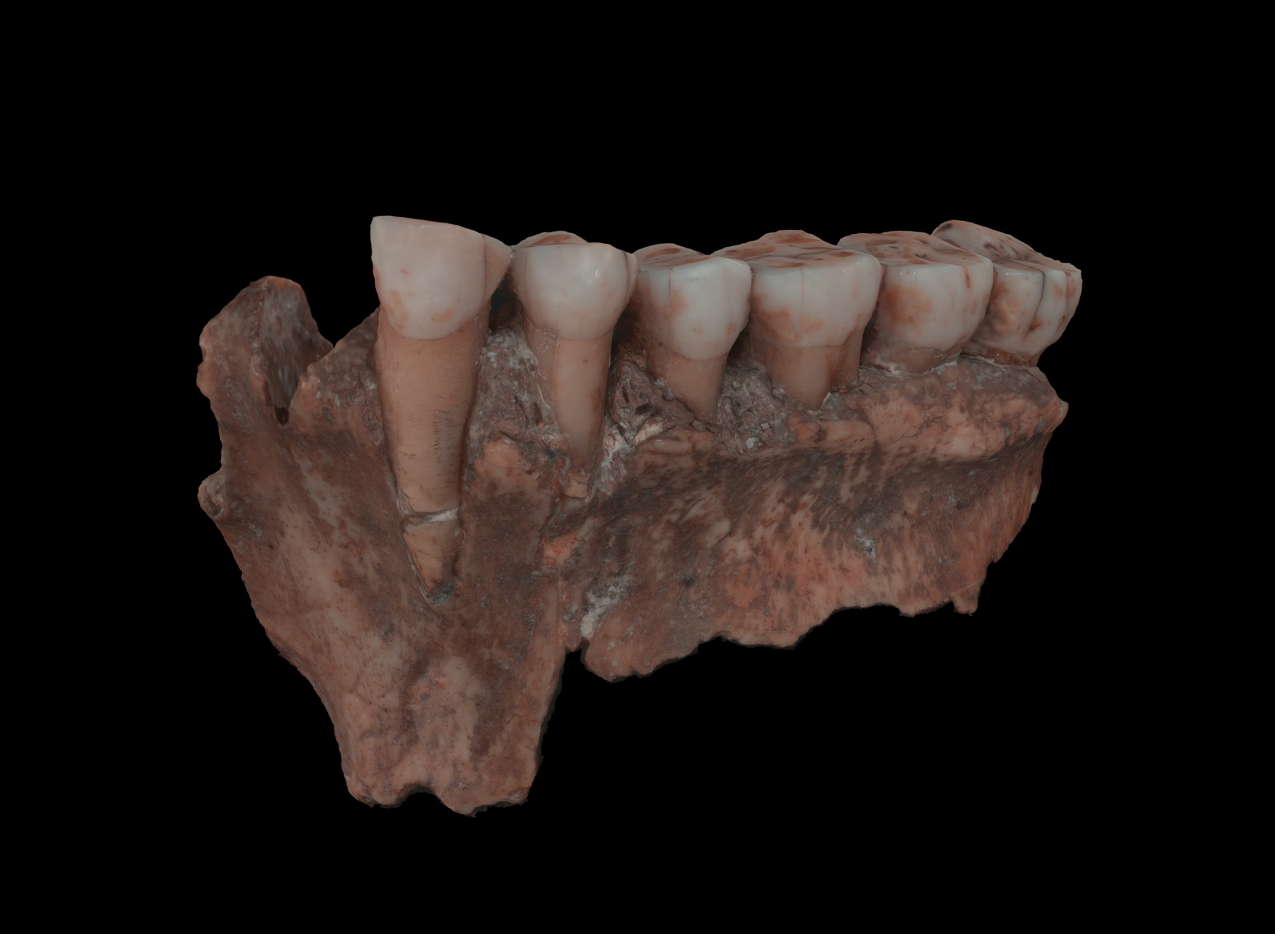
365 nm UVR photogrammetry model with white light texture (https://sketchfab.com/models/3bb1a5a1cde240f3bee18f80c22cb31d/).

For geometrical surface reconstruction, results are consistently the same between human and animal teeth: enamel is best reconstructed in photogrammetry in UV wavelengths, and more specifically in UVR, than in standard white light photogrammetry. Both give better results than the red wavelengths. This phenomenon could be explained by the optical properties of the materials. In order to produce the best model possible of enamel objects, it should be captured in both UVR365 and white light, with pictures taken from the exact same positions. The model should be processed using the UVR pictures and then the white light images can be used to produce a realistic color texture (Fig 34). It is important to use a modified DSLR because the sensitivity of the modified DSLR in UVR is higher than one of an unmodified DSLR.

Even if UVR photogrammetry models are of better quality than the white light models, they aren’t perfect. MicroCT models can give great result with enamel but segmentation and surface extraction can be time consuming in order to avoid artefacts. 3D scanning with structured light gives decent results as well but UVR365 photogrammetry could be less expensive than both techniques at the condition of working with a specific lamp at 365 nm (2 commercial light bulbs at 365 nm are available from 40€) instead of the Megavision light panels used in this study.

Nevertheless, this is a novel method to improve photogrammetry models of challenging materials. The promising results of this study will lead to future investigation on the topic.

Upcoming work will investigate if similar results can be achieved with other challenging materials such as obsidian, alabaster, ivory, quartz, cowries and reflective metals. We will also investigate if digitizing at specific wavelengths can lead to general metric deformations. Additionally, we are developing a low-cost system for spectral/UVR 3D.
